# Constrained Volume Micro- and Nanoparticle Collection Methods in Microfluidic Systems

**DOI:** 10.3390/mi15060699

**Published:** 2024-05-25

**Authors:** Tanner N. Wells, Holger Schmidt, Aaron R. Hawkins

**Affiliations:** 1 Department of Electrical and Computer Engineering, Brigham Young University, Provo, UT 84602, USA; 2 School of Engineering, University of California Santa Cruz, Santa Cruz, CA 95064, USA

**Keywords:** microfluidics, particle trapping, particle enrichment, cell trapping, particle manipulation

## Abstract

Particle trapping and enrichment into confined volumes can be useful in particle processing and analysis. This review is an evaluation of the methods used to trap and enrich particles into constrained volumes in microfluidic and nanofluidic systems. These methods include physical, optical, electrical, magnetic, acoustic, and some hybrid techniques, all capable of locally enhancing nano- and microparticle concentrations on a microscale. Some key qualitative and quantitative comparison points are also explored, illustrating the specific applicability and challenges of each method. A few applications of these types of particle trapping are also discussed, including enhancing biological and chemical sensors, particle washing techniques, and fluid medium exchange systems.

## 1. Introduction

Micrometer and nanometer-sized particles, including both biological and synthetic particles, are incredibly useful subjects for biological and chemical applications. They have been used and studied for use in biosensing, medical diagnostics, and chemical analysis, and their use in these applications and others has become ubiquitous [1,2,3,4]. The ability to contain, concentrate, and control these particles on the micro-scale is similarly invaluable in their use and analysis [5]. Microfluidic platforms are particularly well suited to this role because of the small volumes and dimensions involved in the systems [6].

Enrichment and immobilization of particles into highly constrained volumes has significance in enhancing the capability of tools designed to handle, evaluate, and use those particles. Many types of sensors have detectable limits that may be met or surpassed using dilute samples which have had their concentration enhanced [7,8]. Likewise, many particle processing methods benefit from having subject particles immobilized in small, pre-determined volumes, allowing for more precise control and observation of the particles being processed [9,10].

This review will cover the general techniques used in microfluidic systems to locally aggregate particles of both biological and synthetic origin to enhance their concentration and immobilize trapped particle aggregates. These techniques are presented in Section 2, Section 3, Section 4, Section 5, Section 6, Section 7 and Section 8, and a basic comparison between methods is given in Section 9.

Some applications of these collection techniques will also be discussed, including improvements to microfluidic-based sensors, particle processing, and some other applications [11,12,13]. This paper is not intended to provide a comprehensive review of these applications, but several of them are discussed in Section 9.

Other reviews have been published on related topics, including molecular concentration enhancement [14,15], particle sorting (by size, density, material, etc.) [16,17,18], and single-particle manipulation and trapping, including by optical tweezers [19,20,21,22]. Some reviews have included methods for localized concentration [5,19,23,24]. However, no recent review has been published comprehensively covering methods for collecting micro- and nanoparticles to enhance their local concentration in confined volumes.

For a more comprehensive and detailed background on microfluidics, nanotechnology, biosensors, and nanoparticles, the reader is referred to the following references [6,25,26,27,28,29,30,31,32].

## 2. Trapping Methods Introduction

The principle behind any mechanism for trapping particles is to manipulate the particles independently of their carrier medium in such a way that the particles can be drawn into a confined volume. For this to be accomplished in a microfluidic system, the total force experienced by the suspended particles must be different from that felt by the fluid. Various mechanisms can perform this separation, distinguishing the particles from the suspension medium by size or by optical, electrical, magnetic, or acoustic properties.

A common theme among trapping methods is a size-dependent aspect, where smaller particles are usually less affected by imposed forces than larger ones. One explanation for this is that many of these mechanisms distinguish suspended particles (cells, polymer beads, etc.) from fluid molecules by size. Large particles are trapped by the forces while smaller ones, including carrier fluid molecules, are unaffected.

This size dependence can be troublesome, particularly when very small particles must be concentrated. One example of this is in optical trapping, where optical forces have a strong dependence on particle diameter, proportional to the square or the cube of the particle radius, depending on the force in question [33,34]. Because of this dependence, optical methods such as optical tweezers are usually restricted to manipulating particles in the range of hundreds of nanometers or larger [35]. This is true to varying degrees in most of the particle concentration methods discussed here.

The size dependence of trapping mechanisms also has the potential to be advantageous in some applications. Many of the methods that have been developed for particle trapping have been further studied for size-selective trapping, where particles of one size are collected while other particles follow the flow of the medium [36,37,38]. Desirable effects of this size-dependent trapping include selective filtering of undesired particles or selective enrichment of particles of interest.

In the following sections, particle methods are sorted by the underlying mechanism or force used to trap particles in place. In some cases, multiple effects are utilized simultaneously, and methods that fall into this category have been placed in Section 8.

## 3. Physical Methods

The methods defined here as “physical trapping” are generally passive trapping methods based on the physical manipulation of particles with no external fields applied, except for perhaps pressure on the fluid. These methods can be sorted into two general categories: barrier-based trapping and flow-based trapping.

### 3.1. Barrier Trapping

This method of particle trapping generally implies particles being trapped at a wall or other physical barrier that impedes the movement of particles while allowing fluid to continue passing. Probably the most basic example of this is the porous membrane filter, where particles are trapped on the filter while fluid continues to flow through unfilled perforations in the membrane.

Zheng et al. showed an example of a microfabricated version of the membrane filtration method, where evenly spaced pores allow for faster and more consistent particle trapping on a parylene membrane [39]. This was shown to be particularly advantageous for capturing and enriching tumor cells from a blood sample. This method spreads the enriched sample evenly across a wide area, which may be undesirable or advantageous depending on the application.

Other examples exist of trapping methods that collect particles in an ordered way, often in many individual traps. Wlodkowic et al. created a microfluidic platform with a large array of mechanical traps, again for tumor cell collection, that relies on hydrodynamic effects to trap these cells and shield them from high shear stress [40].

**Figure 1:**
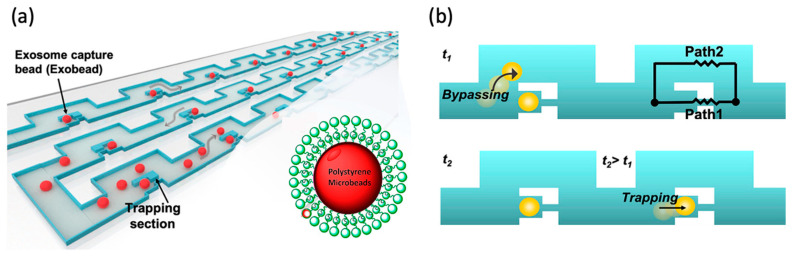
An example of physical trapping of particles into individual trapping sites: (**a**) the hydrodynamic method used to trap these particles is depicted; (**b**) a hydraulic resistance model is shown to describe the trapping effect. This mechanism was used to capture microbeads (red in (**a**) and yellow in (**b**)) with exosomes attached to their surfaces, as depicted in (**a**). Adapted with permission from [41]. Copyright 2020 American Chemical Society.

Similar examples include the works of Tayebi et al. and Tan and Takeuchi, where traps are arrayed along the sides of serpentine microfluidic channels, though in different configurations in the two examples [41,42]. In the first case [41], the traps are placed inside the channel, with a bypass channel around each trap for when it is filled (shown in Figure 1). In the last case [42], the traps take the form of gaps in the walls between serpentine channels so that the traps fill sequentially. Kim et al. expanded the work of Tan and Takeuchi by adjusting the design to increase the number of available trapping positions and make the microparticle trapping size selective [38].

Other methods collect particles in aggregate, usually relying on dual-height channels or similar micro- or nanogaps to trap particles in flow. A few designs have been proposed for this type of trapping.

**Figure 2:**
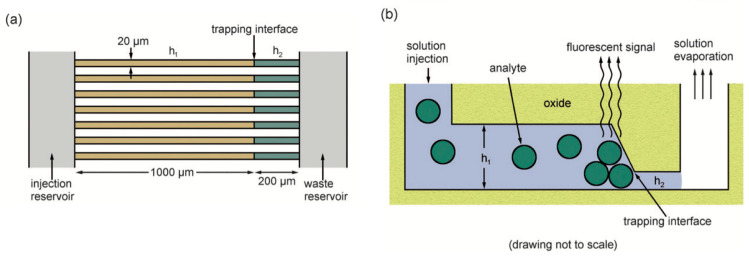
An example of dual-height particle trapping: (**a**) top-view depiction of a series of parallel channels, each with 2 sections of different heights; (**b**) cross-sectional depiction of one of these channels, showing the mechanism used for particle collection. Reprinted with permission from [43]. Copyright 2009 Royal Society of Chemistry.

Hamblin et al. demonstrated dual-height nanofluidic channels capable of size-selective trapping of nanoparticles by trapping the particles at the interface between the two channel heights, as depicted in Figure 2 [43]. This work was expanded to include multiple types of viruses and proteins, and surfactants and AC electric fields were used to decrease undesired clogging [37,44]. Additionally, channels of sequentially decreasing height were used to trap particles and proteins of different sizes into different sections of the channel.

Stout et al. from the same research group presented a similar device, but with electrical actuation that allowed the channel height to be manipulated by an applied voltage [45]. This produces an effect that allows particles to be trapped and released based on an electrical signal.

Dual-height channels were also explored by Tonomura et al. in a device that integrates many nanofluidic channels along the sides of a larger microfluidic channel [46]. This produces relatively high throughput particle trapping that is used to preconcentrate particles for introduction into a sensing micropore.

**Figure 3:**
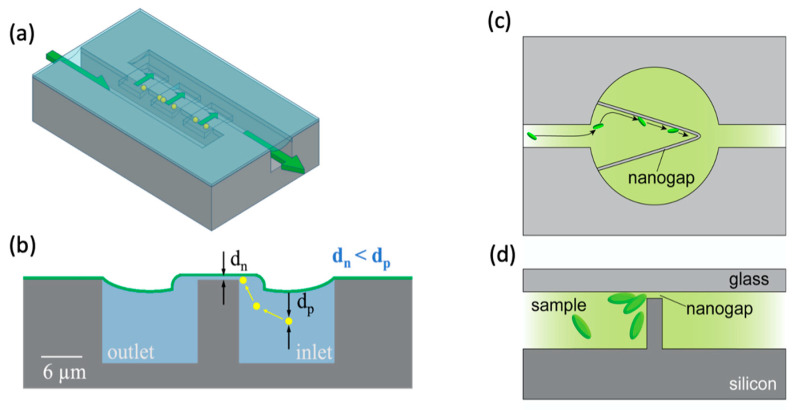
Two related examples of dual-height particle trapping. (**a**) Three-dimensional view and (**b**) cross-section of membrane-covered physical trapping mechanism from Ref [12]; (**c**) top view and (**d**) cross-section of V-shaped physical trapping mechanism from Ref [47]. Reprinted from [12,47] with the permission of AIP Publishing.

A similar concept was developed by Wells et al. for particle concentration in a small volume near a membrane, with potential applications including nanopore sensing [12]. Again, dual-height channels are used, in this case with the smaller nanofluidic channels only along the edges of a small trapping region, as depicted in Figure 3a,b, resulting in constrained trapping.

Another gap-based device was produced by Han et al., this time with a V-shaped wall in a larger channel, the wall being bridged by a nanogap that acts as a trap, as shown in Figure 3c,d [47]. The shape and flow parameters of the trap cause particles to be directed toward the apex of the “V” shape, allowing for high enrichment while retaining high flow rates. The paper is presented for the specific application of concentrating bacterial particles to improve the sensitivity of single-point confocal Raman spectroscopy analysis.

Some tradeoffs are associated with the barrier method of particle trapping. One of these is a balance between flow rate, trapped particle size, and trapping volume. To decrease the minimum size for particle trapping, the smallest dimension of the barrier must be decreased, which causes a substantial increase in flow resistance [48]. The width of the barrier can be adjusted to account for this, but this increases the length over which the particles are distributed, increasing the total trapping volume.

### 3.2. Flow Trapping

The terms flow trapping and flow-based trapping refer here to methods of collecting particles in a location where the fluid flow is stagnant or circulatory. This is possible through multiple effects, but microfluidic flow-based trapping relies almost exclusively on vortex-based particle aggregation.

Aggregation of solid matter in fluid vortices is a relatively intuitive and well-known phenomenon. Some widely observed examples of the collection of matter in flow eddies include leaves or branches being drawn into a whirlpool in a stream, or a tornado trapping and carrying debris within its vortex. In microfluidic devices, fluid dynamics can be precisely controlled to create vortices where particles can be collected within micrometers of a desired location.

There are numerous methods for creating these flow patterns on a microfluidic scale, including electrical, thermal, and geometry-based techniques. This section will focus solely on those influenced only by geometry, with no need for externally applied fields other than a pressure differential. Vortex trapping using other mechanisms, like flow-generating electrical effects, will be treated later in this paper.

**Figure 4:**
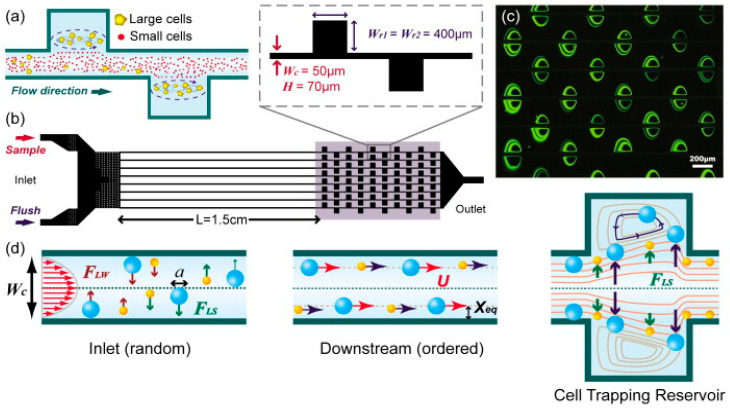
Flow-based trapping mechanism from [36]. (**a**) Selective trapping of large particles is depicted; (**b**) a top view of the channel design; (**c**) a top view showing fluorescent particles trapped in vortices; (**d**) a depiction of the underlying selective trapping principle. Reprinted from [36] with the permission of AIP Publishing.

Much of the current vortex-based trapping research stems from the work of Hur, Mach, and Di Carlo [36], where it is shown that a protrusion on the side of a microfluidic channel produces a microscale vortex that collects particles from the flow, as shown in Figure 4. This effect is dependent on particle size, and larger particles are preferentially trapped while smaller ones pass through the device. Overall efficiency and throughput are improved by placing many of these traps along the sides of multiple channels. These devices were then used to selectively separate and trap circulating tumor cells from whole blood [49].

More recent work with the devices demonstrated particle enrichment with a shear-thinning carrier fluid, which was shown to enhance the size-based enrichment effect [50]. More recently, there have been studies on the movement of particles trapped in the vortex flows showing that particles can follow repeated single- or dual-orbit patterns [51,52].

An example of flow-based particle trapping not reliant on vortex generation was recently presented by Kwon et al. [53]. In this size-selective trapping method, it is shown that spiral-shaped inertial microfluidic channels, often used for size-based particle separation, produce a trapping effect along the edge of the channel for particle sizes that approach the width of the channel.

## 4. Optical Methods

Light can act on matter in various ways to influence particle trajectories. Light reflected or absorbed by an object imparts momentum to that object, a phenomenon referred to as optical radiation pressure or scattering force [54]. Further, many microparticles act as lenses, changing the angle of photon movement and causing an exchange of momentum between light and matter. In a uniform optical field, the lensing forces generally balance to produce no net force, but a gradient in the field results in a force on the particle that can be used to direct it. The magnitude of the force is proportional to the optical field gradient, so the phenomenon is referred to as the gradient force [55]. These forces have been used in various ways for particle manipulation. Paiè et al. and Cai et al. both wrote recent reviews on ways that optical particle manipulation methods have been implemented in microfluidic systems, including to collect particles [19,56].

When discussing particle trapping by optical methods, the technique that most commonly comes to mind is that of optical tweezers. These high-precision tools are extremely powerful for trapping and manipulating single particles. Due to the great success of optical tweezers, optical trapping methods are most often applied for single particle or single cell manipulation. However, various optical techniques have been developed or adapted for larger-scale particle trapping and accumulation.

Some of the methods adapted for multiple particle collection are based on optical tweezer effects. An example is time-sharing optical tweezers, like those developed by Mirsaidov et al. which were used to create a stable array of 200 *E. coli* cells, demonstrating the ability of optical tweezers to create structured cell collections analogous to living tissue [11]. Another example of adapted optical tweezers is the refractive multiple optical tweezers implemented by Werner et al. that stably trapped more than 200 yeast cells in flow [57]. An advantage offered by these optical tweezer-based methods is that trapped particles or cells may be individually manipulated by changing the position of each beam. However, this often comes at the cost of complexity and lower enrichment factors.

**Figure 5:**
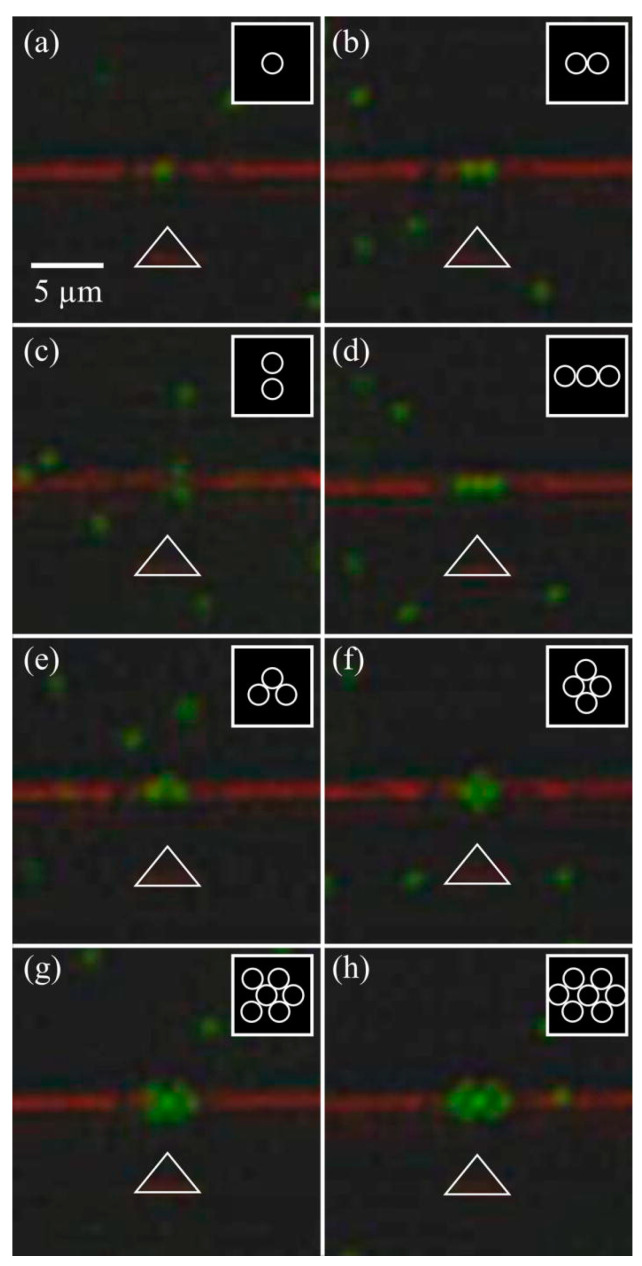
(**a**–**h**) Structured particle trapping by a photonic crystal cavity. The hollow triangles point to the trapped particles, and the insets with hollow circles show the trapped particle structure. Reprinted from [59] with the permission of AIP Publishing.

Other techniques exist for structured optical manipulation of particles. Mandal et al. demonstrated a 1D photonic resonator that was used to trap particles in a line [58]. Subsequent research by Renaut et al. produced a similar device that used a photonic crystal cavity to assemble microparticles into structured configurations dependent on the number of particles trapped, as shown in Figure 5 [59].

Kang et al. demonstrated a device with ordered sets of microantennae, each of which is capable of trapping a single particle using optically induced vortex forces [60]. This vortex-based approach was shown to be more effective at trapping smaller particles than many other optical methods.

While these methods of structured particle manipulation provide opportunities for more precise control over individual particle placement, particle concentrating efficiency is again often sacrificed to achieve this structuring. In the context of this paper, these structured methods are generally less effective as means of particle concentration than many other methods. Some more efficient examples of optical particle aggregation from dilute suspensions follow.

**Figure 6:**
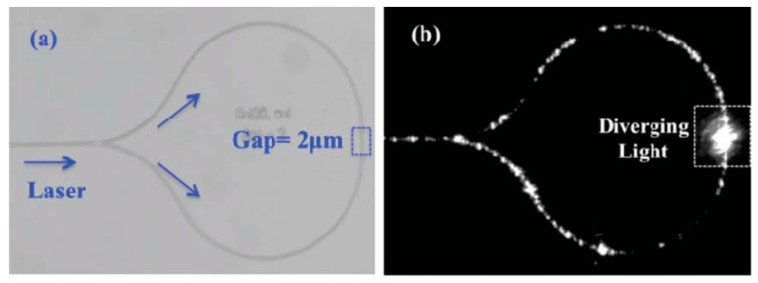
(**a**,**b**) Two counter-propagating waveguides attract particles using optical gradient forces from the evanescent field near the waveguide, then direct these particles to a collection point using optical radiation pressure. Reprinted with permission from [62]. Copyright 2012 Royal Society of Chemistry.

An interesting phenomenon relating to particle interactions with waveguides was recorded in 1996 by Kawata and Tani [61]. The researchers observed that particles near an optical fiber would be influenced by the evanescent optical field extending from the waveguide. The effect of this interaction was that particles would be attracted to the waveguide and, once very close to the fiber, drawn along the fiber in the direction of light propagation. This phenomenon was explored as a method of localized particle immobilization, and later Hellesø et al. demonstrated a microfluidic-based device that could collect many particles in a predetermined volume [62]. In this device, two counter-propagating, on-chip waveguides provide radiation pressure to guide particles to a predefined region at a gap between the ends of the two waveguides, where the particles become trapped between the two rapidly diverging, counter-propagating beams. The design and particle trapping are shown in Figure 6.

Another example is the work of Sergides et al. where plasmonic resonance—resonance of free electrons in a metal when stimulated by an optical field—was used to overcome the size limitation of many other optical methods, allowing them to trap large numbers of nanoparticles [63].

Kühn et al. produced another method of particle aggregation in a microfluidic setting [64,65,66,67]. Two counter-propagating beams are fed into a liquid-core channel from integrated waveguides. The opposing beams trap particles and hold them in place, similar to [62]. In this example, however, the optical beam is contained within the liquid channel using an anti-resonant reflecting optical waveguide (ARROW) structure, allowing particle-carrying fluid to continue flowing through the channel, delivering particles to the trapping region.

**Figure 7:**
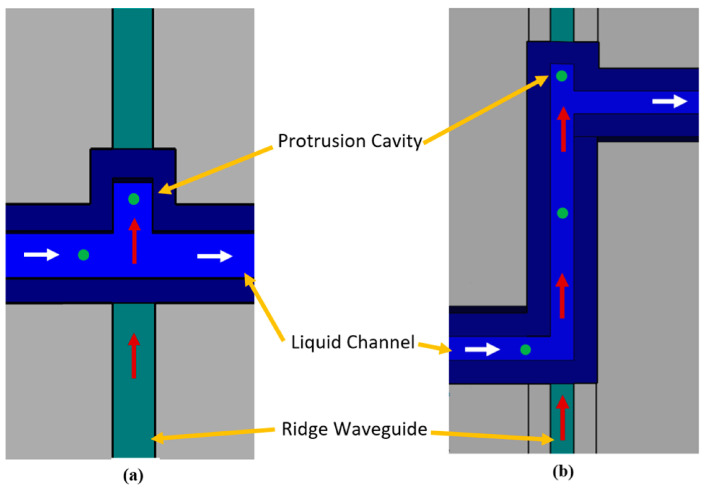
Optical particle trapping into a protrusion in an enclosed fluid channel using (**a**) optical radiation pressure and (**b**) a combination of optical radiation pressure and optical gradient force, in both cases provided by an integrated waveguide. Reprinted from [34] under open access Creative Commons CC BY license.

From the same group, Walker et al. demonstrated a similar method of trapping particles in a liquid channel using optical effects [33,34]. In this work, light is fed through an on-chip, solid-core waveguide into a liquid-core channel. As particles flow through the channel, optical gradient and radiation forces guide and push them into a geometrically defined protrusion where the particles are collected, as depicted in Figure 7. The particles are trapped near a thin membrane spanning the channel top; the membrane may serve as a platform for a sensor such as a nanopore [8,68].

## 5. Electrical Methods

While electric fields are best known for their interactions with charged particles, as in ubiquitous electronic devices and systems, various electrical effects can also be used to manipulate particles of neutral or near-neutral charge, a category that includes most biological and synthetic particles. One example is dielectrophoresis (DEP), a technique in which a strong field gradient induces electrical polarization in a particle, allowing nonuniform electric fields to impart energy to the particle. In addition, some electrical phenomena, such as electro-osmosis and the electrothermal effect, can generate fluid movement which, if properly controlled, can induce particle aggregation.

### 5.1. Dielectrophoresis

By far the most common electrical method for particle collection is dielectrophoresis (DEP). The principle of DEP can be explained as follows: while a neutral dielectric particle is unaffected by a uniform electric field, a nonuniform field of sufficient strength can act on the induced polarity in the particle, allowing it to be manipulated by the field. The direction of the force on the particle depends on the properties of the particle and of the suspending fluid, as well as on the frequency of the AC electric field [69]. If the force is in the same direction as the applied field, then this is termed positive DEP (pDEP), while a particle that experiences a force in the opposite direction is said to be subject to negative DEP (nDEP). In both cases, this method has been used to great success to manipulate individual particles and to act collectively on large groups of particles.

Herbert A. Pohl first defined the term dielectrophoresis in relation to the observation that strong, inhomogeneous electric fields cause particle motion and aggregation on an electrode [70]. The configuration used in his example consisted of two simple electrodes, a tungsten wire, and a ring of tin foil, placed in particle-carrying liquid in a Petri dish. He observed that when a 10,000-volt AC or DC potential was placed on the electrodes, carbon particles would quickly migrate to the negative electrode.

Early microfluidic examples of DEP trapping were demonstrated by the groups of Huang et al. and Schnelle et al. in the 1990s [71,72,73,74]. These showed that both synthetic and biological particles could be collected using relatively simple planar electrodes. The success was such that numerous patents were issued based on the method [75,76,77].

**Figure 8:**
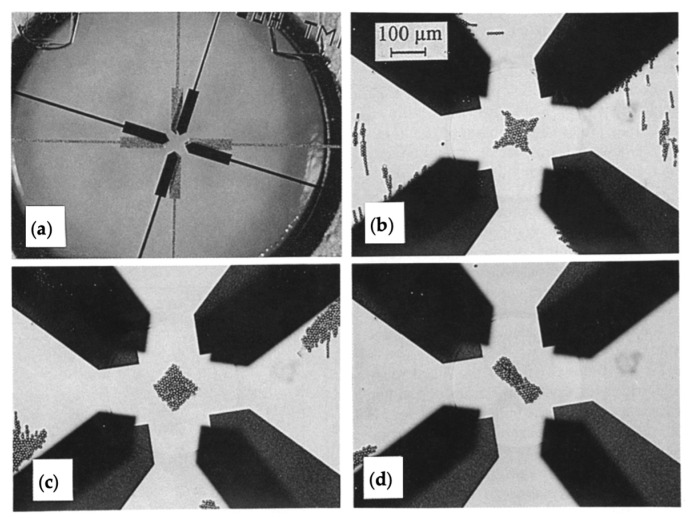
A dielectrophoresis-based particle trapping and manipulation device. (**a**) A top-down picture of the device, showing 4 upper electrodes and 4 lower ones; (**b**) particles trapped in suspension, out of contact with the device surfaces; (**c**,**d**) the shape of the trapped particle cluster can be manipulated by changing the electrical phase difference between the alternating current signals of the separate probes. The scale is equal for (**b**–**d**). Reprinted in part from [72], with permission from Elsevier.

Most of these early methods are based on a four-electrode design, where the polarity of electrodes can be switched to allow for positive or negative DEP [71]. Schnelle et al. used a similar concept, but with eight electrodes at two separate heights to manipulate the three-dimensional field gradient, allowing for non-contact trapping and more precise manipulation [72]. The device and resulting particle trapping and manipulation are shown in Figure 8.

An important variation on dielectrophoresis, including for trapping, is insulator-based DEP (iDEP). This method uses insulator geometries, rather than electrodes, to produce the magnetic field gradients necessary for dielectrophoretic particle manipulation. Lapizco-Encinas wrote a somewhat recent review on iDEP [23], so related methods will not be covered extensively here. However, some articles more specifically related to particle trapping using the iDEP method are referenced.

Most iDEP devices use an electric field placed across a channel, while constrictions designed into the channel enhance the field gradient locally. A fascinating example of this comes from the work of Masuda et al., who were the first to report on an iDEP system [78]. In their paper, they report trapping cells using electrode-free DEP forces, which then bring the cells together. Their result led to a now commonly used method for inducing cell fusion, an important process in modern biological research [79].

Some more recent developments have helped to advance the use of iDEP in particle trapping. Nakidde et al. developed insulator-based DEP devices that use three-dimensional geometries to locally enhance electric field gradients, allowing for favorable trapping efficiency at higher flow rates [80]. Similarly, Chiou et al. produced iDEP channels with nano-scale constrictions, enhancing field gradients to a point where the devices could trap nanoparticles [81].

High-frequency alternating currents are typically used to mitigate the potential for other effects to interfere with DEP trapping. These other effects include electrolysis, which can generate gas bubbles, and electrokinetic effects that can cause undesired fluid movement. However, direct current dielectrophoresis (DC DEP) has some advantages that have maintained its presence in research, including finer control of particle movement [82].

Insulator-based DEP is generally less sensitive to DC-specific issues like electrolysis due to much larger distances between the electrodes and the trapping region. For this reason, most DC DEP is performed in conjunction with iDEP.

Early studies of insulator-based DC dielectrophoresis were performed by the group of Lapizco-Encinas, Cummings, and Singh [82,83]. They demonstrated trapping of polystyrene beads and biological particles in a channel with a simple two-electrode DC configuration. Insulated posts in the channels are used to enhance the localized electric field gradient between the posts, leading to particle aggregation near the posts. This aggregation was also shown to be selective between different types of bacteria due to their differing dielectric properties.

Chen and Du used a different configuration for DC DEP, in which the field gradient is enhanced at channel junctions [84]. The result is a channel with multiple trapping sites, capable of collecting 930 nm polymer particles.

A major problem with the DEP method of particle trapping is that the method only collects particles in localized areas of high field gradients, often in the direct vicinity of the electrodes. This means that particles far from these areas are unaffected, and thus cannot be trapped. Additionally, the trapping forces are often too weak to overcome fluid drag force even if low flow rates are used to pass particles near the trap. For these reasons, the aim of much of the research involved with dielectrophoretic trapping has been to increase the affected volume by various means.

**Figure 9:**
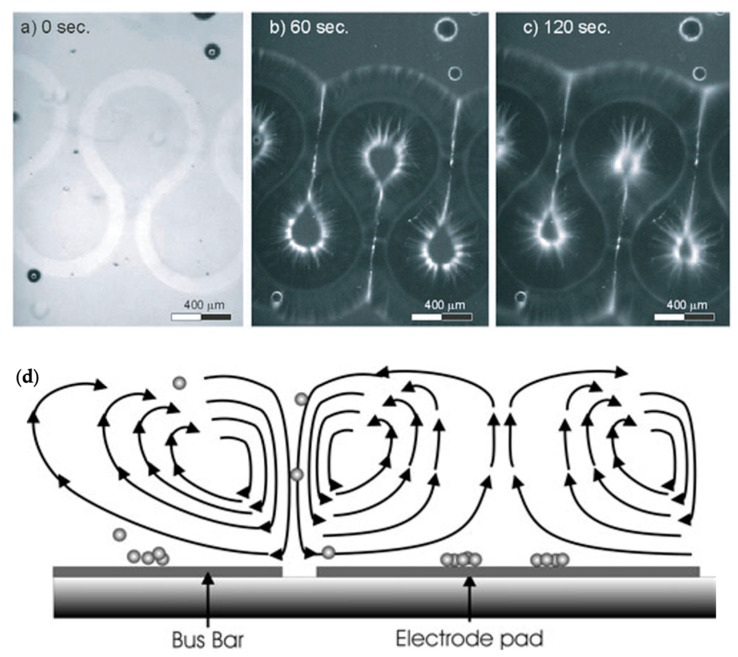
Particle trapping by dielectrophoresis, enhanced by electro-osmotic flow. (**a**) A brightfield image of the electrodes; (**b**,**c**) a fluorescence image of particles collecting on the electrodes as time progresses. Particles collect on the electrode pads due to DEP, and vortices produced by ACEO (depicted by the circulating arrows in (**d**)) allow wide-scale circulation of particles to enhance the range of the trapping effect. Reprinted with permission from [85]. Copyright IOP Publishing. All rights reserved.

Hoettges et al. developed so-called “zipper electrodes” that took advantage of electrohydrodynamic forces, most notably alternating current electro-osmosis (ACEO), an electrokinetic phenomenon that causes bulk fluid movement in electrolytic fluids [77,85,86]. They showed that at frequencies of ~1 kHz, the fluid movement from these electrodes was strong enough to induce fluid circulation in a wide area, bringing particles to the electrodes where they could be captured by DEP, as shown in Figure 9. The same group further characterized this method to show that trapping is largely independent of particle size, expanding the potential applications to a wide variety of biological particles, and even carbon nanotube alignment [13,87].

Wong et al. demonstrated another particle trap design combining ACEO, DEP, and other electrokinetic forces [88,89]. The devices described by this group were used to trap a wide variety of biological particles, greatly enhancing their concentration. They showed the method’s effectiveness at trapping particles of a wide range of sizes, ranging from single-strand DNA as short as 20 base pairs (single-digit nanometer dimensions) to *E. coli* bacteria (1–2 µm dimensions).

Gagnon and Chang again combined DEP with ACEO, this time using much higher AC frequencies [90]. In their paper, they showed that ACEO could be used to quickly move fluid towards the DEP trap, significantly enhancing trapping speed compared to that reported by previous methods.

Cheng et al. also used a combination of DEP and ACEO forces to rapidly concentrate particles, this time with the specific intent of improving surface-enhanced Raman spectroscopy (SERS), a useful biological analysis tool that relies on high localized concentrations of analytes [91]. The authors also showed that AC frequency variation resulted in the separation of red blood cells from bacteria, another useful enhancement for SERS.

Another mechanism that has been used to supplement DEP is the alternating current electrothermal effect (ACET). ACEO and ACET are closely related phenomena, as they are both generated by AC electric fields in the fluid medium. However, ACEO uses flow generated by the movement of ions in an electric field, while ACET uses flow generated by thermal effects due to Joule heating of the liquid medium.

ACEO is generally better understood and more frequently utilized, but ACET can have some unique advantages, especially in media with higher electrical conductivity [92]. Park et al. demonstrated a useful example of this type of trapping, where ACET currents move particles to a DEP trap [92]. They further predicted which electrokinetic effects would dominate particle movement in a range of AC frequencies and fluid conductivities.

### 5.2. Electrokinetic Fluid Trapping

“Electrokinetic” is a term that describes the movement of fluid or particles that either results from or results in the generation of an electric field [93]. This section describes electrokinetic effects that cause particle carrier fluid to move in such a way that the flow field traps particles.

While electrokinetic effects like alternating current electro-osmosis and electrothermal effect (ACEO and ACET) are often used in conjunction with DEP to enhance the latter’s performance, there are other means of collecting particles using these phenomena.

Electro-osmosis is a phenomenon in which an applied electric field generates bulk fluid movement in small-scale systems, like capillaries or microfluidic channels [88]. It relies on the formation of an electric double layer (EDL) at the liquid–solid interface. An applied voltage produces charge movement in the liquid, thus inducing flow. Electro-osmotic flow can be generated using either direct current or alternating current electric fields. In microfluidics, direct current electro-osmosis is most frequently applied as a fluid driving mechanism, as an alternative to pressure-driven flow [94]. Alternating current electro-osmosis is more frequently used for microfluidic particle trapping applications than its direct current variant [86,95].

**Figure 10:**
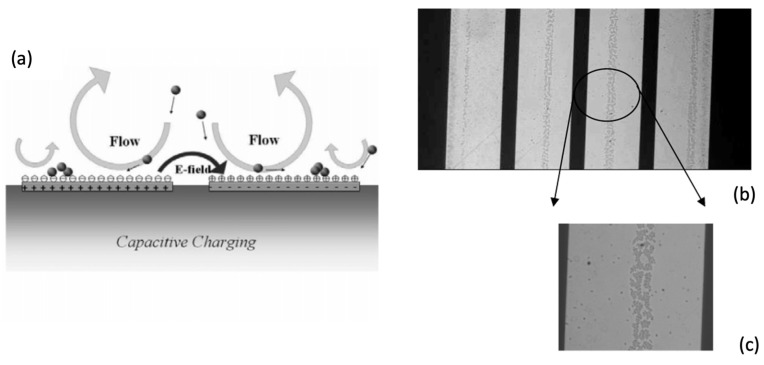
Alternating current electro-osmosis (ACEO) particle trapping using induced vortex flows. (**a**) A depiction of vortex flows generated by ACEO; (**b**,**c**) *E. coli* particles collected in a line on the center of electrodes. Adapted with permission from [96]. Copyright 2005 American Chemical Society.

Trapping designs based on ACEO generally rely on vortex flows generated by AC electro-osmosis to stagnate particles, much like the vortex flows used by Hur et al. and the other methods described in Section 3.2 [36]. Wu et al. demonstrated an example of this, showing that relatively simple use of ACEO forces can cause particles to aggregate in lines on an electrode surface, as shown in Figure 10 [96]. They provided further insight into ACEO particle trapping by describing optimal conditions for trapping synthetic and biological particles in structured lines on electrodes, also showing that particle detection by impedance measurements can be performed in such a system [97].

Bhatt et al. showed another configuration for electrical trapping, mainly attributed to ACEO [98]. In this case, an AC electric field is placed vertically across a fluid-filled chamber, with an electrode spanning the top of the chamber and a patterned electrode on the bottom. Particles collect on the patterned lower electrode, and the effect of field strength and frequency on particle velocity is described.

Another example of ACEO trapping was given by Hou et al., where vortex flows concentrate particles into a small volume for SERS analysis, an application that was mentioned in Section 5.1 [99].

Dey et al. gave further insight into this method of trapping with a device consisting of a channel with a constricted section, across which an AC field was placed [100]. The device was shown to trap charged particles in a symmetric AC field. The effects of frequency variation on trapping were studied, with different mechanisms (ACEO, electrophoresis, and positive and negative DEP) present in different frequency ranges.

The alternating current electrothermal effect (ACET) was mentioned in the previous section as a method of enhancing DEP trapping. Similarly to ACEO, this effect can also be used independently to trap particles using vortex flows. ACET relies on the generation of thermal gradients by joule heating of the carrier fluid, inducing large-scale flow.

**Figure 11:**
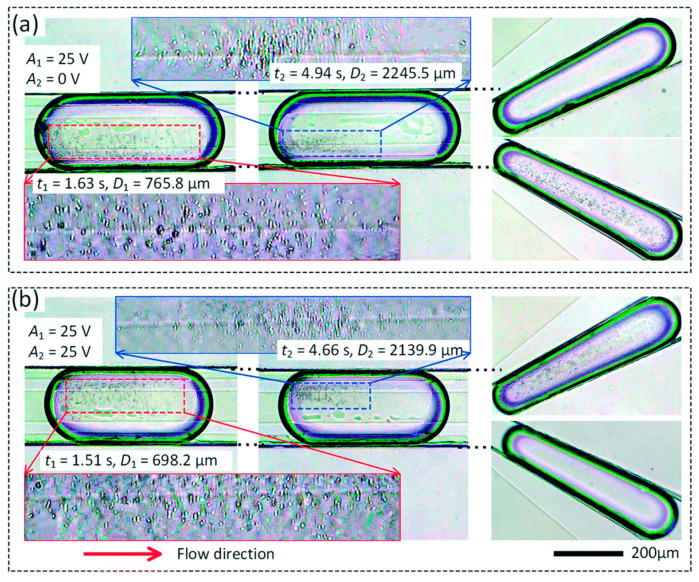
Particle collection using the alternating current electrothermal effect (ACET). Vortices generated by ACET cause particles to migrate (**a**) to the bottom of a droplet or (**b**) to the top of a droplet. The droplet can then be divided to separate particle-carrying fluid from particle-free fluid. The magnified images indicated by the arrows in (**a**,**b**) show the distribution of particles during processing (red) and after particles have been concentrated (blue). Reprinted with permission from [103]. Copyright 2021 Royal Society of Chemistry.

Yang and Wu showed through numerical simulations that ACET and ACEO can both be used to concentrate particles into small regions through careful manipulations of the flow fields produced by these methods [101,102]. Sun et al. provided a demonstration of the use of the ACET effect to create vortices that captured particles in stagnant regions, all within a single droplet, as shown in Figure 11 [103]. Further, Abdelghany et al. varied electric field frequency to tune electrokinetic trapping (including both ACEO and ACET) to enhance particle trapping efficiency [104].

### 5.3. Other Electrical Methods

A few other electrical effects aside from those discussed to this point have been presented as means for particle trapping.

As early as the 1980s, AC fields were observed to cause micro-scale aggregation of charged particles into coherent crystalline-like monolayers on an electrode surface [105]. A description of the underlying theory behind this effect was given a decade later, explaining that electrohydrodynamic (EHD) flows cause this particle migration and aggregation, overcoming the electrostatic repulsion of similarly charged particles [106,107]. It was also shown that this migration and aggregation occurs under DC and AC electric fields, but not at AC frequencies above 1 MHz.

**Figure 12:**
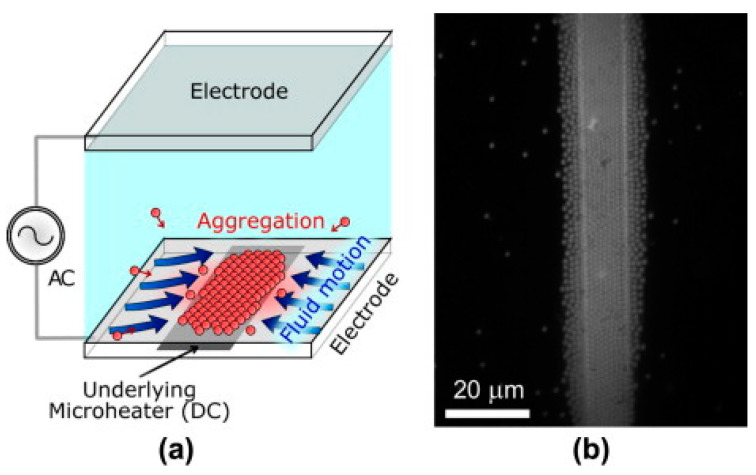
Particle collection onto a heated section of an electrode. (**a**) A depiction of the forces acting on particles to cause them to collect; (**b**) a top-view image of particles collected in a monolayer above a heater. The formation of a monolayer of particles is due to electrohydrodynamic flows from an electrode and is further induced by localized heating of the electrode. Reprinted from [109]. Copyright 2012, with permission from Elsevier.

Further work was conducted by the group of Williams et al., showing that both light and heat can help to induce this effect [108,109]. Because of this, patterned particle monolayers can be formed on electrode surfaces. Heat-induced monolayer formation is shown in Figure 12.

Another device using electrical methods was presented by Guan et al., showing a DEP-like quadrupole trap for charged particles [110]. This trap was shown to have a wider frequency regime than would be feasible if DEP were the main actor. ACEO was also ruled out as the sole contributor by frequency range. It was proposed that a combination of these effects was at play, allowing particles to be trapped at wide frequency ranges.

A final electrical method was shown by Aïzel et al. [111], in which an ion-selective nanochannel caused repulsion and concentration effects to enrich nanoparticles in the channel. Similar methods have been used in many cases for enriching ions in solution, but their applications for larger particles and cells are limited [14].

## 6. Magnetic Methods

The use of magnetic fields is a common method of microfluidic particle manipulation due to the relative ease of generating a field that strongly affects particles and the option to generate the magnetic field externally, reducing design and fabrication complexity. In fact, some examples use only a permanent magnet and a capillary tube for particle trapping [112,113], demonstrating the simplicity that can be achieved in a magnetic trap design.

While magnetic trapping is easiest to perform with magnetic particles, most commonly superparamagnetic beads (SPMBs), this type of trapping greatly limits the types of analytes that can be collected. However, techniques exist for trapping diamagnetic or nonmagnetic particles, including cells and polymer spheres. Most commonly, this involves suspending the sample in a paramagnetic fluid, either ferrofluid or a solution with paramagnetic ions. When exposed to a magnetic field, the difference in magnetism between particles and carrier fluid produces an effect sometimes referred to as “magnetic buoyancy”, allowing the particles to be influenced separately from their medium [112].

In an early example, Watarai and Namba showed that a magnetic field could be enhanced near a capillary to produce field gradients strong enough to trap multiple red blood cells from suspended flow in a capillary [113].

**Figure 13:**
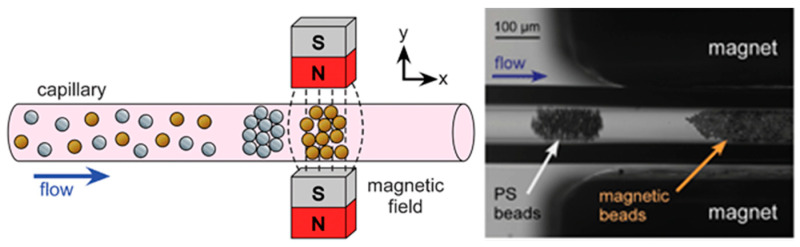
A method of trapping magnetic and diamagnetic polystyrene (PS) beads in a capillary using two external permanent magnets. Particles are suspended in a paramagnetic solution to allow for the trapping of diamagnetic particles. Reprinted with permission from [112]. Copyright 2021 Royal Society of Chemistry.

One of the simplest designs for trapping both paramagnetic and diamagnetic particles simultaneously was demonstrated by Tarn et al. [112]. In this design, a magnet is placed on each side of a capillary tube, while superparamagnetic beads and diamagnetic polystyrene particles flow through the capillary, both suspended in a solution of paramagnetic Mn^2+^ ions. The effect is the creation of two distinct regions of particle aggregation, or plugs, as shown in Figure 13. One plug was shown to consist exclusively of the polymer beads, while the other was shown to only be comprised of the magnetic particles.

Hejazian and Nguyen later created a similar device, this time with an array of permanent magnets on either side of the capillary channel [114]. Particles of two sizes (3.1 µm and 4.8 µm diameters) are introduced into the channel, suspended in dilute ferrofluid. The effect is a size-selective trapping of particles, where larger and smaller particles are each trapped in distinct plugs in the capillary. The trapping characteristics were further evaluated with multiple ferrofluid concentrations and flow rates to better model the behavior of particles in these systems.

Another example, from Kimura et al., also using an externally generated field, showed that modulated magnetic fields could be used to pattern particles and cells on the microscale, trapping them into a repeating series of lines [115,116].

Ramadan et al. presented an example using an on-chip electromagnet [117]. Their research demonstrated that adding a ferromagnetic pillar to the device could increase the local magnetic field gradient, greatly enhancing the trapping efficiency of this method.

**Figure 14:**
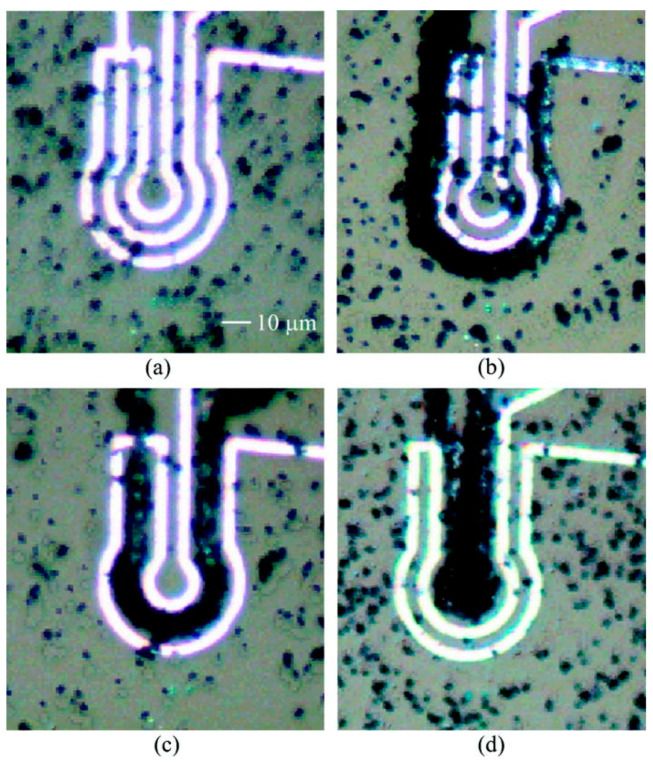
A method of both collecting and sensing magnetic particles using an integrated electromagnet. (**a**) Suspended particles are at first unaffected; (**b**–**d**) voltages are sequentially applied to the outer, middle, and inner ring electrodes to attract particles and move them to the device center. A magnetic sensor is placed at the center of the device, allowing particles to be evaluated. Reprinted from [119], with the permission of AIP Publishing.

Further research on magnetic particle trapping using integrated electromagnets was presented in multiple papers by the research group of Gooneratne et al., with a focus on magnetic-based particle analysis and detection [118,119,120]. In these papers, a set of concentric conductive loops are designed to produce a magnetic field gradient that collects particles from a relatively wide area into a much more confined central point. One example of this is shown in Figure 14. This wide-area particle aggregation may be most useful in systems with little fluid movement, such as droplet-based systems.

The same group also presented a permanent-magnet-based design, where a field from an external bar magnet was used [121]. The field gradient was enhanced using a series of microfabricated ferromagnetic micropillars, producing an ordered formation of trapped magnetic particles.

**Figure 15:**
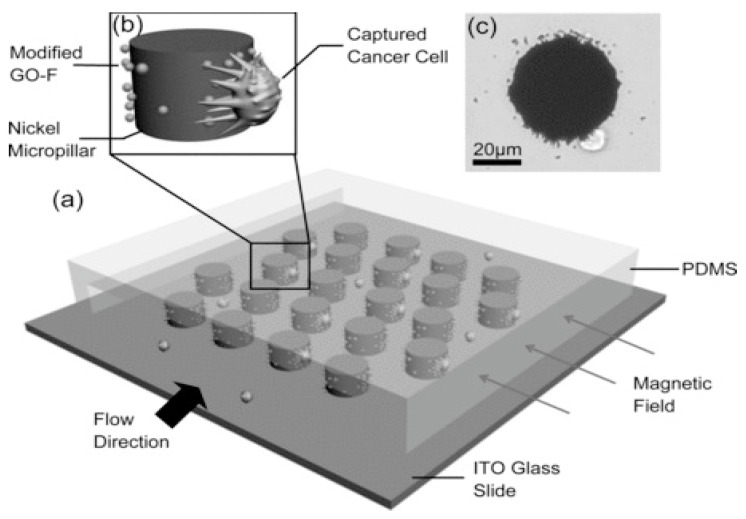
An external magnetic field-based device for selective localized cell capture. The device uses an external magnetic field that couples to ferromagnetic pillars, enhancing the local magnetic field gradient and attracting surface-modified magnetic nanoparticles. These particles in turn capture cancer cells on their modified surfaces. (**a**) A depiction of the trapping device; (**b**) a depiction of a single pillar capturing a cell; (**c**) a microscope image of a cell trapped on a pillar. Reprinted with permission from [122]. Copyright 2013 WILEY-VCH Verlag GmbH & Co. KGaA, Weinheim, Germany.

Yu et al. used a device design very similar to that of [121], but with some extra steps for a more specific application [122]. The group presented a method of coating iron oxide nanoparticles with a graphene oxide layer, and then functionalizing their surfaces for specific cell capture. The micropillar chips were then used to trap these surface-modified magnetic nanoparticles, which in turn were used to immobilize cancer cells for testing and later release. The cancer cell trapping is depicted in Figure 15.

Another pillar-based device was presented by Faivre et al., in which the device was created from an iron–PDMS (polydimethylsiloxane) composite material [123]. This material choice retains many of the numerous advantages afforded by PDMS for microfluidic systems, while simultaneously allowing for the creation of magnetic structures for creating high magnetic field gradients on-chip. The shape of the pillar structures was adjusted to produce high field gradients and trapping efficiencies.

A significant issue associated with devices using integrated electromagnets is Joule heating resulting from high current densities in microfabricated coils. Researchers have been able to address this issue by various means.

Smistrup et al. increased the thickness of the conductors and added integrated cooling channels to a chip to both reduce and remove generated heat [124]. Later, Lefebvre et al. optimized an electromagnetic coil for high trapping efficiency with minimal waste heat, and the trap was used to collect 300 nm magnetic particles [125].

Some groups have even used the heat generated by coils for advantageous effects, most notably to create an optimal temperature for applications with living cells. Song et al. used Joule heating and microfluidic coolant channels to maintain a steady temperature in the sample channel [126], and Zheng and Sawan simply adjusted the current in the coil to control temperature for a similar effect [127].

## 7. Acoustic Methods

Acoustic waves have been used to manipulate particles, including to trap and levitate water droplets or other matter, for nearly a century [128]. A common example of acoustic waves for particle aggregation is in an experiment used to demonstrate the behavior of waves. In this demonstration, sand is dispersed onto a plate, and acoustic standing waves in the plate cause the sand particles to aggregate onto the nodal lines induced by the standing waves.

In microfluidics, applications of acoustic particle manipulation have been more recent, and research is ongoing. There are two main acoustic forces that produce particle manipulation effects in microfluidics, namely acoustic radiation and acoustic streaming [24].

The principle behind the acoustic radiation force is that an acoustic wave traveling through a particle-containing medium can impart its energy into the particles due to differing acoustic properties between the particles and the medium [129]. This is similar to light diffracting and imparting momentum when traveling between materials of differing refractive indices.

Acoustic streaming is a separate phenomenon, wherein bulk fluid flow is induced by an acoustic oscillation. This can be caused by multiple effects but generally can be attributed to an imbalance in fluid forces as the fluid is pushed and pulled by the oscillatory wave [25].

The acoustic radiation force is capable of precise particle manipulation, including immobilization and trapping. Acoustic streaming can be used to create vortex flows in the microfluidic system, which can be used to generate fluid circulation or to induce particle trapping.

An example of microfluidic particle trapping based on acoustic effects was demonstrated by Lilliehorn et al. [130]. In this research, an array of individually addressable ultrasonic transducers directly beneath the sample is used to perform the trapping. Due to high field gradients near the transducers, each transducer creates a separate particle trapping region, immobilizing beads above the element while fluid continues to flow.

**Figure 16:**
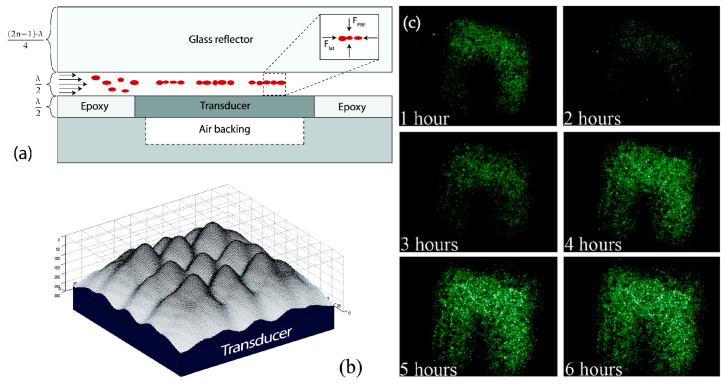
Acoustic trapping and holding of cells during a 6 h cell culture. (**a**) A cross-sectional depiction of the device; (**b**) a depiction of the pressure distribution produced by the acoustic transducer; (**c**) an immobilized cell cluster is observed through the course of a 6 h culture experiment. Reprinted with permission from [10]. Copyright 2007 American Chemical Society.

A related paper by the same research group studied the effect of this acoustic trapping on bioparticles and living cells [10]. They showed that cells trapped in this acoustic field could be cultured while the medium around them continued to flow, as shown in Figure 16, providing opportunities for culture medium exchange without significantly disturbing the cultured cells.

Many of the applications for acoustic particle collection involve the manipulation of particles within a single droplet, an outcome that is difficult for many other trapping methods. An example of this is the work of Shilton et al., who successfully concentrated 500 nm particles within a single droplet using vortex flows generated by surface acoustic waves, which also provided a mixing effect [131].

The same group and some others continued to use this phenomenon to precisely manipulate particles within single droplets. Raghavan et al. provided insight into the underlying mechanism by modeling the three-dimensional trapping behavior and producing experimental supporting results [132]. The group also demonstrated a size-dependent aspect to this trapping, in which particles of two sizes (6 µm and 31 µm) were independently concentrated within the droplet by controlling the input acoustic power [133].

Destgeer et al. provided further insight into droplet-based acoustic trapping, evaluating the particle collection using multiple testing parameters [134]. They concluded that this method produces four distinct particle concentration regimes, in which particles are collected into different volumes in the droplet. The regime applicable to any given experiment was shown to be dependent on acoustic wave frequency and amplitude and on the particle size used.

Whitehill et al. demonstrated a slightly different approach to acoustic trapping in a droplet, in that much lower frequencies were used: tens of Hertz rather than the typical MHz [135]. The results were produced with larger particles than previous methods, and they showed that there are again distinct trapping regimes dictated by particle radius and acoustic frequency.

**Figure 17:**
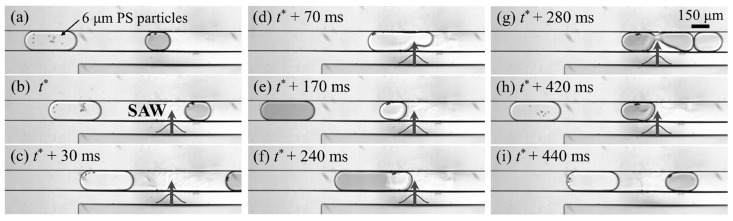
Particle enrichment and medium exchange in a droplet through a surface acoustic wave (SAW). (**a**) A droplet with 6 µm particles is shown entering the processing region; (**b**,**c**) particles collect in a confined region of the droplet due to the SAW; (**d**) the droplet is cut, increasing particle concentration; (**e**) the new suspension medium is introduced; (**f**) the particles are moved to the new medium; (**g**) the new droplet is cut, again enriching the particles; (**h**,**i**) the process repeats. Reprinted with permission from [9]. Copyright 2018 Royal Society of Chemistry.

A final example given here of droplet-based acoustic particle trapping is that of Park et al., who presented a method that combines enrichment and washing onto a single device [9]. First, particles are suspended in a droplet inside of a microfluidic channel. Forces generated by surface acoustic waves are used to collect the particles into a confined region on one side of the droplet. These forces are also used to cut the droplet, reducing the total volume of carrier fluid, and thus increasing the concentration of particles within the droplet. Washing is performed by the same mechanism, where the droplet with particles and the droplet of new suspension medium are combined, the particles are pushed into the new medium, and the droplet is cut, all before the media have time to intermix. This is shown in Figure 17.

Small particles can be much more difficult to trap, especially at high flow rates. Hammarström et al. addressed this by introducing particle clusters into the trapping region, which act as a seed to induce further particle aggregation [136]. Similarly, Evander et al. used larger particles as seed particles to collect much smaller biological microparticles from a small volume (10–100 µL) of blood plasma [137]. In this latter example, the particles were both collected and washed in the same platform.

Another method of trapping smaller particles is by simply increasing the actuation frequency of the acoustic field. This can be difficult beyond the MHz frequency range, but Cui et al. demonstrated enrichment of sub-100 nm particles by use of a gigahertz range acoustic trap [138].

Zhou et al. showed that sub-micron particles could be captured using acoustic streaming produced by low-frequency acoustic waves [139]. In their paper, they used 800 Hz vibrations, coupled with micropillars in the channel, to produce vortex flows near the pillars that successfully collected 800 nm particles.

**Figure 18:**
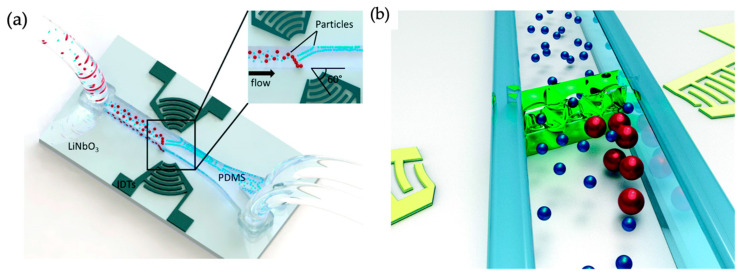
Particle filtration using an acoustic “membrane”. Large particles are selectively trapped by the surface acoustic wave, enriching them at the trapping location. (**a**) An expanded depiction of the acoustic trapping channel with larger (red) particles becoming trapped and smaller (blue) particles unaffected; (**b**) a magnified depiction of the trapping region showing the virtual membrane (green) trapping larger (red) particles but allowing smaller (blue) particles to pass. Reprinted with permission from [140]. Copyright 2016 Royal Society of Chemistry.

The group of Fakhfouri et al. demonstrated multiple platforms that produced size-dependent trapping of microparticles [140,141]. In the first, a “virtual membrane” produced by surface acoustic waves focused into a plane in the channel was shown to affect different sizes of particles (in this case, 7 µm and 10.4 µm particles) differently depending on the acoustic power level, depicted in Figure 18. In the second, the acoustic waves caused a streaming effect, producing vortices in the channel that preferentially collected larger 2 µm particles and allowed most smaller 1 µm particles to pass through.

## 8. Other Methods

The previously described methods are the most common ones for particle collection in microfluidic systems, but other techniques exist, including hybrid methods that use multiple effects to enhance particle trapping in a variety of ways. Some hybrid methods were discussed in previous sections (e.g., optically induced vortices for nanoscale particle trapping), and some more will be treated here.

A trapping method that has been shown to be useful for trapping cells is chemical-based trapping through channel surface modification. This method has classically been used for the patterning of proteins on a surface, but specific cell trapping has also been performed using this technique [142]. This is achieved by first applying cell-specific antibodies to the channel surface, usually in a patterned way. When the targeted cells are introduced into the channel, they bind to the antibodies, while other analytes pass through [143,144]. Mu et al. covered some variations of this method in a 2012 review [145].

Various hybrid methods have been proposed and demonstrated, combining advantages from separate methods to achieve more optimal trapping. In some cases, this serves to enhance trapping efficiencies or flow rates, and in some others, it allows for the trapping of smaller particles.

Sigurdson et al. demonstrated a device in which the alternating current electrothermal effect (ACET), described in more detail in Section 5.3, is used to generate vortex flows, which direct particles towards a surface with chemical binding sites [146]. This was shown to enhance the rate at which particles can be trapped.

Another hybrid method, this time combining physical and electrical methods, was shown by Syed et al. [147]. A standard physical trapping mechanism with nanofluidic filter channels is used to trap a large group of silica nanospheres, effectively clogging the larger sample channel and forming a stable structure. This porous structure is then used for electrokinetic concentration of protein and DNA samples.

Gerspach et al. also combined physical and electrical methods to produce a device intended for trapping nanoparticles [148]. The group developed a method of geometry-induced electrostatic (GIE) trapping, based on electrostatic repulsion between charged particles and a similarly charged channel, causing particles to migrate to a predetermined point. The group used pneumatically controlled channels to change the channel geometry at will, allowing for easily controllable trapping and releasing of particles. This method produced arrays of individually trapped single particles, as opposed to aggregations of particles.

Another physical–electrical hybrid method was shown by Krafft et al. [149]. Their device contains two channels separated by a porous membrane microfilter. One channel supplies the sample: in this case, one containing pathogenic bacteria. The other channel simply serves as an electrical conduit. As the sample flows past the membrane in the first channel, electrokinetic flow produced by a strong electrical potential across the membrane carries particles to the membrane to be trapped.

**Figure 19:**
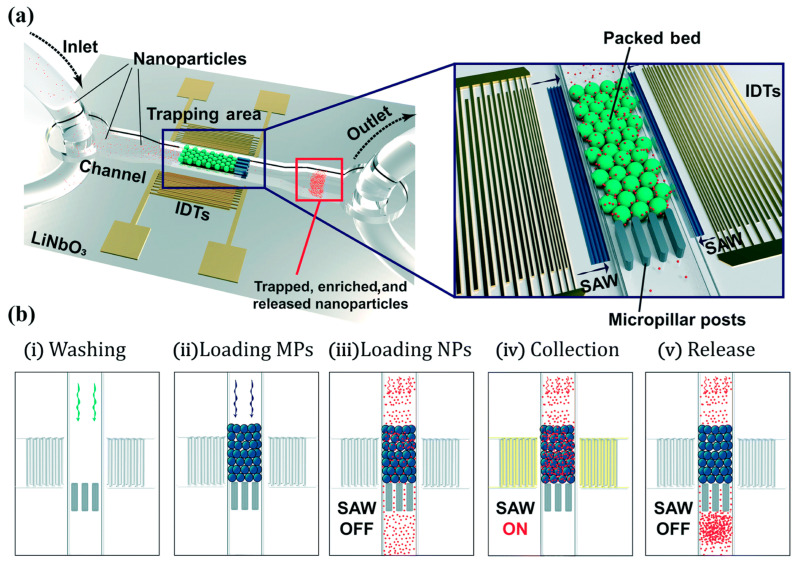
A hybrid particle trapping method that combines physical barrier-based trapping of larger 10 µm particles with acoustic trapping of much smaller nanoparticles. This allows the surface acoustic waves (SAWs) to capture much smaller particles than would otherwise be feasible. (**a**) A depiction of the particle trapping device showing the packed bed of larger (green) particles and acoustically trapped smaller (red) particles; (**b**) a depiction of the trapping mechanism showing physical trapping of larger (blue) particles and acoustic trapping and release of smaller (red) particles. Reprinted with permission from [150]. Copyright 2019 Royal Society of Chemistry.

Habibi and Neild used a combination of physical and acoustic trapping to collect nanoparticles that would be more difficult to trap by either method individually [150]. First, 10 µm particles are physically trapped by posts in the channel, forming a bed of trapped particles. Then, an acoustic transducer is activated, and 500 nm particles are trapped between the larger beads due to a high acoustic field gradient between the large particles, as depicted in Figure 19.

Allahrabbi et al. demonstrated that dielectrophoresis could be combined with surface modification to enhance the collection effects of both [151]. In this case, a DEP mechanism directs cells to a chemically modified surface, where they are trapped by non-specific binding.

Other hybrid methods were reviewed by Kumar et al. and Al-Ali et al. [152,153]. Not all these methods are applicable to particle trapping—some are specific to separation of particles by size or other characteristics. However, exploration of these reviews could yet be useful for research into combinations of particle manipulation mechanisms that have previously been explored.

### Comparison

While each of the discussed trapping methods has many characteristics and features, each of which may or may not be desirable for a given application, there are a few points on which the methods may informatively be compared. These include flow rate, particle size (or size range), localized trap volume, and trapping efficiency.

One way of combining many of these metrics into a single value is through the enrichment factor (EF), which may be defined as the ratio of the final localized concentration of particles ($\chi_{f}$) to the initial concentration ($\chi_{i}$),

(1) $${EF}_{1}=\frac{\chi_{f}}{\chi_{i}}.$$

Many articles report an enrichment factor directly, while others report values that can be used to calculate the enrichment factor, such as initial concentration, flow rate, size of the trapping volume, and number of trapped particles. Some relations can be derived to calculate the enrichment factor based on these other values. For example, because the final localized concentration of trapped particles ($\chi_{f}$) is simply a ratio of the number of trapped particles ($n_{trapped}$) to the trap volume ($V_{trap}$), we can rewrite Equation (1) as

(2) $${EF}_{2}=\frac{n_{trapped}}{V_{trap}\chi_{i}},$$

And because the number of trapped particles can be expressed as a product of the total number of particles that flow through the device ($n_{total}$) and the trapping efficiency (η), or the proportion of particles that become trapped,

(3) $${EF}_{3}=\eta \frac{n_{total}}{V_{trap}\chi_{i}}.$$

Consider the fact that the total number of particles that enters the trap is the product of the total volume of the particle-carrying fluid that enters ($V_{total}$) and the initial concentration of particles in that fluid ($\chi_{i}$), and we see that

(4) $${EF}_{4}=\eta \frac{V_{total}}{V_{trap}}.$$

In many cases, the total volume that flows through the trap is not reported, but the flow rate (Q) is. In this case, we may express Equation (4) in terms of time (t) as

(5) $${EF}_{5}=\eta \frac{Q\;t}{V_{trap}}.$$

It must be noted that for Equations (4) and (5) to be valid, η must represent the *external* trapping efficiency of the device, or in other words the total proportion of particles introduced into the device which also are collected in the trapping region. In cases where some particles never enter the vicinity of the trapping region, for instance, if particles become trapped in an inlet reservoir or channel, then the concentration of particles reaching the trap will be different from the concentration introduced into the device, and the concentration terms will not cancel to produce Equation (4) unless the efficiency factor takes this into account. An example of this is shown in Ref. [12], in which the authors stated that approximately half of the particles became stuck in an inlet reservoir, never making it to the trap. The authors reported a trapping efficiency of “100% efficiency for particles that reach the trap”, or a 100% *internal* trapping efficiency, which would correspond to about a 50% external efficiency, accounting for the approximately 50% of particles reportedly trapped in the reservoir.

While the enrichment factor can be a useful metric, it must be considered alongside other parameters to be meaningful for comparison. One example is the amount of time taken to perform the particle concentration. Many particle trapping processes have a linear relationship between enrichment factor and time, as represented in Equation (5); if trapping volume, efficiency, and volumetric flow rate are constant with time, then the enrichment factor is directly proportional to the time. So, the longer a test is run, the higher the enrichment factor will be.

Furthermore, particle size must be considered when comparing methods by enrichment factor. For example, the physical trapping method reported in Ref. [47] produces an enrichment factor close to 10^6^ in 10 min for approximately 1 µm particles. However, the method relies on a size-based trapping approach, meaning that if smaller particles were used, the channel geometry would need to be adjusted to trap these particles, which would in turn reduce the flow rate and thus the enrichment factor for a given time. In other examples, trapping efficiency and trap volume can be affected by particle size.

Another important consideration is the flow rate used, the total volume, or the number of particles processed. In some cases, an increased flow rate could enhance the enrichment factor, while in others such enhancement is prevented by other constraints, like the balance between the trapping force and the drag force on the particle. Additionally, many methods are only able to process a small volume because of either flow rate constraints or a limit to the total number of particles able to aggregate at a time.

Some quantitative comparison points are shown in Table 1, with a single reference for each of the general trapping mechanisms. Each reference was chosen because many of the pertinent values were either given in the paper or could be found from data in the paper and because enrichment factors were relatively high compared to those reported in other papers using the same mechanism.

Many of the important comparison points between trapping methods are not easily quantifiable. These include whether a method is active or passive, whether it traps particles in a permanent or a reversible manner, whether the particles are collected in a structured way, and the cost and complexity of the device design, fabrication, and use. These qualitative attributes are compared in Table 2.

Some comparison criteria are dependent on other factors aside from the trapping technique used. For example, many of the methods can be implemented with devices constructed from a variety of materials—a device may be constructed from silicon or from polydimethylsiloxane (PDMS), a choice which could simultaneously affect fabrication cost and device performance [154].

The first row of Table 2 shows the attributes used as qualitative comparison criteria for the trapping mechanisms shown in the first column. A description of each attribute follows:

- Passive: no external forces or fields are used for particle trapping.
- Structured: captured particles can be arbitrarily arranged into multiple configurations.
- Non-contact: particles do not come into direct contact with the solid portion of the device.
- Reversible: particles or cells may be freely released after capture.
- Size-selective: mechanism selectively traps particles in a specific size range.
- Complexity (fabrication): a general measure (low, medium, or high) of how complex the trapping platform is to initially fabricate, based on complicating factors such as small dimensions, a large number of process steps, and expensive fabrication facilities.
- Complexity (use): a general measure (low, medium, or high) of how complex the trapping platform is to implement, based on complicating factors like expensive equipment, active particle tracking, and the need for precise control mechanisms.
- Enrichment capability: a general measure (low, medium, or high) of the ability of a trapping mechanism to locally concentrate particles, including flow rate, enrichment factor, etc. Some numerical substantiation is given in Table 1.

No single trapping method will be perfect for all possible applications. The tables are provided as a simplified means of comparing these methods from a high level, but a deeper understanding of each mechanism and of the hypothetical application must be acquired before a thorough comparison can be made.

## 9. Applications

Microfluidic particle collection methods have found many applications, most of which are in some way related to biology, (bio)chemistry, or medicine. The basic ability to process a dilute sample of cells or other particles into a constrained volume with high concentration can greatly enhance the sensitivity and ability of techniques intended to process or analyze those particles. In some cases, it may open avenues to analysis or processing methods that would otherwise be ineffective or impossible.

Once an aggregation of particles has been trapped in place, the particles may then be analyzed or treated using various methods. Most of the articles referenced in this review cite specific applications in particle processing or sensing. These include biosensors and bioassays, Raman spectroscopy, particle medium exchange, surface modification, and tissue engineering. These specific applications will be discussed in greater detail here. Other applications that will not be discussed in detail include precision drug delivery [138], cell fusion [78], and cancer-specific analysis [39,40,49,122,143,144].

### 9.1. Detection Enhancement

An example of a sensing method that benefits from a high localized particle concentration is Raman spectroscopy. Raman spectroscopy is an analysis technique in which the spectrum of light scattered off a sample can be used to evaluate chemicals, identify compounds, and even provide insight into molecular structure and interactions [7]. The technique has substantial applications in biological analysis and other fields, but spectrum analysis can be difficult due to the Raman scattering effect being quite weak [47]. Physical, optical, electrical, acoustic, and hybrid particle trapping methods have all been shown to concentrate particles in a way that can improve this analysis technique [47,62,91,99,149,155].

Another example of a particle analysis method that can greatly benefit from locally concentrating particles is the nanopore sensor. A nanopore consists of a nanometer-scale hole in a membrane through which a sample flows. A voltage is placed across the pore, causing an ionic current to flow through the pore. This current changes as a particle passes through the nanopore, and the change in current can be used to detect and perform basic analysis on the particle. This method can be used for DNA or RNA sequencing or for counting and basic analysis of particles, including virus sensing [156,157].

A major problem associated with nanopore sensors is the low flow rate through the pore. A meaningful volume of sample must pass through the pore for complete analysis to take place, and flow rates through a pore with a diameter on the order of nanometers can be extremely slow. For analysis to be completed in a reasonable time, the concentration of analytes in the sample must be very high, or a very large number of pores must be placed in parallel and individually monitored. Particle collection near a pore can considerably improve the sensitivity of the method, allowing it to be used for much more dilute samples than would be possible otherwise [68,158,159,160].

A few groups have used particle collection methods to improve nanopore sensors in this way. Most of these were addressed previously in the trapping methods sections, and they included barrier-based and optical particle trapping [8,12,33,34,46,68].

Other examples of particle sensing techniques that can be enhanced by collecting particles in a predetermined region include surface-based techniques like plasmon resonance [63,87,95] and visual techniques like colorimetry [161].

The complete range of sensor enhancement applications of microfluidic particle trapping techniques has likely been explored only superficially. Particle collection in a confined volume can be very powerful in analytical settings, and this is a promising area for future work in the field.

### 9.2. Particle Medium Exchange

In addition to sensing applications, the ability to immobilize cells in a constrained volume can also be a valuable tool for the preparation of biological and synthetic samples. This can take various forms, but a common application is in the exchange of sample suspension media.

Many sample preparation methods for particle suspensions require the suspension medium for these particles to be exchanged at one or multiple points in the preparation process. The steps where this medium exchange is performed are collectively referred to as washing [9].

Particle washing can be performed using various methods, including dilution, centrifugation, or filtration on a perforated membrane. Microfluidic trapping methods have at least one distinct advantage when it comes to washing: they are able to hold particles in place while the medium is exchanged, in many cases using non-contact methods, leading to greater sample observability, more thorough medium exchange, and very low particle count losses during washing steps. An example of particle washing using trapping techniques was shown previously in Figure 17.

A closely related application is in the continuous exchange of a cell culture medium while a culture experiment is occurring. Multiple techniques have been shown to provide the capability to hold cells in a constrained volume while the surrounding medium is exchanged and to do so in a way that keeps the cells viable and does not interfere with their reproduction [10,36,126,127,162]. Particle trapping used in conjunction with this method also allows the cells to be closely monitored during culture. A visual example of this was shown earlier in Figure 16.

### 9.3. Surface Modification and Labeling

**Figure 20:**
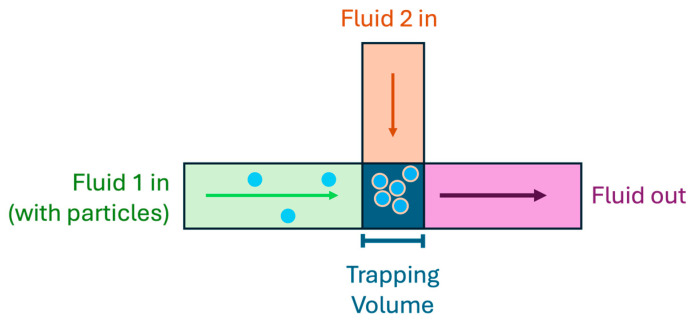
An example setup for a particle processing application. Particles suspended in fluid 1 flow into the trapping volume and are collected. Fluid 2 could be either a new medium for the particles (in the case of washing) or another solution, perhaps containing a particle dye or labelling molecule. Excess or used fluid is removed through the outlet channel.

Another application that may use similar techniques to particle washing is that of particle surface modification. Modification of particle surfaces may occur for a few different reasons, but most commonly it is performed as a means of particle labeling, either for fluorescence microscopy or for immunoassays [163,164,165]. A diagram of an example setup for particle surface modification is shown in Figure 20.

An interesting demonstration of this was given by Tarn et al., in which the authors tested the ability of a magnetic particle trap to hold particles in place during a chemical reaction on the particle surfaces [112]. First, paramagnetic and diamagnetic particles were trapped using a permanent magnet-based technique. The particles formed two clusters, one each comprised of the paramagnetic and diamagnetic particles. In a first test of the reaction capability, the diamagnetic particles were tested using a streptavidin–biotin binding assay with a fluorescent dye bound to the biotin molecules, resulting a visible increase in the fluorescence of those particles, while the paramagnetic beads remained non-fluorescent. The second test was equivalent, but with the particle types reversed, resulting in fluorescence of the paramagnetic particles.

In addition to showing that the particles were selectively trapped as intended, these tests also demonstrated that particles could be collected, labeled, and washed while trapped in a single constrained location. Some other examples demonstrated a similar ability to label particles in situ [166,167].

### 9.4. Tissue Engineering

Another application that has employed particle trapping techniques is that of tissue engineering. This field of study involves manipulating cells to place them in a precise pattern, the goal of which is to emulate biological tissue. While most of the particle trapping techniques discussed in Section 2, Section 3, Section 4, Section 5, Section 6, Section 7 and Section 8 were presented as methods of collecting particles in aggregate, with no predetermined pattern, a few are capable of this precise manipulation [11,168,169].

This application is distinct from the previously discussed applications in that its success depends on the pattern in which cells are placed, while the others require particles only to be immobilized and locally concentrated. Table 2 shows that two of the generalized methods provide inherently structured trapping, while four of the other methods may be configured for this type of application.

## 10. Conclusions

Enhancement of particle concentration in a localized volume can be an extremely valuable tool in systems designed to analyze, manipulate, and otherwise act on the particles. This review covers the range of microfluidic-based techniques that have been used to collect particles for this purpose.

These techniques rely on physical, optical, electrical, magnetic, acoustic, and some other forces to keep particles contained. The technique used, as well as other variables such as material choice, particle composition, and size scale, can affect how the trapping takes place. Complexity, trapping volume, flow speed, and enrichment factor are a few of the trapping characteristics that depend on these chosen variables.

Many of the applications for microfluidic particle trapping and concentration relate to sensor enhancement or sample preparation. Many sensing methods can be enhanced by increasing local concentrations of the particles to be sensed, and many of the methods discussed here excel in this regard. Sample preparation using these techniques often relies on immobilizing suspended particles while the suspension medium is changed or refreshed. This provides the opportunity to observe particles held in place while chemical reactions or other processes are performed on the particles. With many of these methods, the particles may be subsequently released after the sensing or alteration has been performed.

Recent developments have been made in many particle collection methods in the last few years, including physical [12,47,53], optical [8], and electrical [104] mechanisms. Mechanisms like dielectrophoresis that have a long standing in microfluidic systems have the distinct advantage of well-established fabrication and use processes. However, in practical use settings, newer passive mechanisms with low use complexity will likely be of greater interest for potentially wide-spread applications like medical diagnostics. With these methods, tradeoffs may include higher fabrication complexity, lower reusability, or lower enrichment capability.

Particle collection research is ongoing, and much work may yet be performed both in the implementation and application of these methods. The full variety of mechanisms that may be used for particle immobilization has likely not been fully explored. Further, the range of applications in which these methods may be of benefit has great capacity for expansion. More research into combining multiple trapping methods may prove beneficial for some applications, and further integration of trapping systems into particle processing and analysis systems could allow for greater use of the methods.

While biosensor enhancement and biological analysis have been some of the main targeted applications for these systems, their widespread implementation in practical use settings is still limited, as is the case with many lab-on-a-chip systems [170,171,172]. Further work to simplify and lower the cost of these systems, especially with respect to their ease of use, may allow for their broad commercial realization.

Even given the possibility for future work, the current state of the research shows that both developing and well-established methods can be used to great effect for a variety of important applications. The field of microfluidic particle trapping for localized enrichment has great potential in the years to come for biological and chemical sensing, analysis, sample preparation, and more.

## Figures and Tables

**Table 1:** Quantitative comparison of trapping mechanisms. A single reference is chosen for each trap type based on a relatively high enrichment factor for a given process time.

Reference	Trap Mechanism	Particle Type, Diameter	Flow Rate (µL/min)	Trapping Efficiency	Trap Volume	Initial Concentration (particles/mL)	# of Trapped Particles	Enrichment Factor	Time	Notes
[38]	Physical	Bacteria ~0.5–2 µm	10		~5 pL *	1000 CFU/mL	~100 CFU	~10^6^ *	10 min	
[25]	Optical	Polystyrene 1 µm	6 × 10^−4^	98%	1 pL *	4 × 10^7^	~140	~3600 *	12 min	These values were taken from the results for the design with higher efficiency
[63]	Electrical	Bacteria ~1 µm	12	90%	~10 nL *	10^9^		~1000 *	1 min	The values were from a high enrichment case
[94]	Magnetic	Diamagnetic; paramagnetic 10 µm; 8 µm	0.2	100%	~1 nL *	10^6^	~1000 *	~1000	~10 min	Paramagnetic particles could be trapped at higher flow rates, but not diamagnetic ones
[124]	Acoustic	Cancer cells 15–20 µm	4	~100%	~4 nL *			~1000	1 min	A high rate, high enrichment case was used

* These values were not reported in the paper; they were instead calculated from reported and estimated values in the paper.

**Table 2:** Qualitative comparison chart for trapping mechanisms. Each trapping mechanism (row) is assessed to determine to what degree it possesses each property (column). A green check indicates that the mechanism inherently has the given property, a red X indicates that the mechanism cannot possess the property, and an orange circle indicates that the mechanism may or may not possess the given property, depending on the configuration.

		Passive	Structured	Non- Contact	Reversible	Complexity (Fabrication)	Complexity (Use)	Enrichment Capability	References
**Physical**	Individual sites	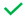	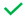	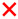	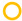	Medium	Low	Low	[38,39,40,41,42]
	Dual-height channel	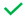	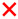	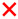	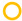	High	Low	High	[12,43,44,45,46,47]
	Inertial microfluidics	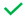	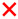	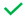	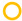	Medium	Low	Medium	[36,49,50,51,52]
**Optical**	Multiple optical tweezers	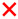	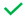	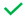	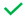	Low	High	Low	[11,57]
	Integrated waveguide	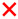	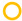	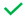	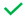	High	Medium	High	[8,33,34,66,67]
**Electrical**	DEP	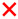	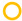	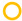	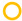	High	Medium	High	[71,72,73,74,85,86,87,88,89,90,91,92]
	iDEP	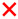	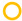	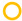	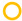	Medium	Low	High	[78,80,81,82,83,84]
	Electrokinetic flow	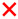	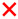	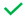	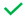	Medium	Medium	Medium	[96,97,98,99,100,101,102,103,104]
**Magnetic**	External magnet	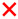	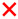	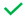	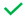	Low	Low	Medium	[112,113,114,115,116]
	External magnet, micropillars	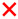	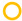	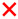	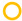	Medium	Low	High	[121,122,123]
	Integrated electromagnet	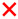	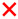	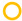	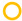	Medium	Medium	High	[117,118,119,120,124,125,126,127]
**Acoustic**	ARF	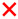	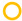	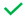	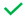	High	High	High	[9,10,130,134,136,137]
	Acoustic streaming	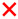	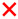	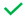	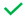	High	Medium	Medium	[131,132,133,134,135,141]

## References

[1] Yin H., Marshall D. (2012). Microfluidics for Single Cell Analysis. Curr. Opin. Biotechnol..

[2] Gholizadeh S., Shehata Draz M., Zarghooni M., Sanati-Nezhad A., Ghavami S., Shafiee H., Akbari M. (2017). Microfluidic Approaches for Isolation, Detection, and Characterization of Extracellular Vesicles: Current Status and Future Directions. Biosens. Bioelectron..

[3] Li W., Zhang L., Ge X., Xu B., Zhang W., Qu L., Choi C.-H., Xu J., Zhang A., Lee H. (2018). Microfluidic Fabrication of Microparticles for Biomedical Applications. Chem. Soc. Rev..

[4] Khizar S., Zine N., Errachid A., Jaffrezic-Renault N., Elaissari A. (2022). Microfluidic-based Nanoparticle Synthesis and Their Potential Applications. Electrophoresis.

[5] Nilsson J., Evander M., Hammarström B., Laurell T. (2009). Review of Cell and Particle Trapping in Microfluidic Systems. Anal. Chim. Acta.

[6] Sackmann E.K., Fulton A.L., Beebe D.J. (2014). The Present and Future Role of Microfluidics in Biomedical Research. Nature.

[7] Hoffmann G.G. (2017). Raman Spectroscopy, Volume I: Principles and Applications in Chemistry, Physics, Materials Science, and Biology.

[8] Sampad M.J.N., Saiduzzaman S.M., Walker Z.J., Wells T.N., Wayment J.X., Ong E.M., Mdaki S.D., Tamhankar M.A., Yuzvinsky T.D., Patterson J.L. (2024). Label-Free and Amplification-Free Viral RNA Quantification from Primate Biofluids Using a Trapping-Assisted Optofluidic Nanopore Platform. Proc. Natl. Acad. Sci. USA.

[9] Park J., Destgeer G., Kim H., Cho Y., Jin Sung H. (2018). In-Droplet Microparticle Washing and Enrichment Using Surface Acoustic Wave-Driven Acoustic Radiation Force. Lab. Chip.

[10] Evander M., Johansson L., Lilliehorn T., Piskur J., Lindvall M., Johansson S., Almqvist M., Laurell T., Nilsson J. (2007). Noninvasive Acoustic Cell Trapping in a Microfluidic Perfusion System for Online Bioassays. Anal. Chem..

[11] Mirsaidov U., Scrimgeour J., Timp W., Beck K., Mir M., Matsudaira P., Timp G. (2008). Live Cell Lithography: Using Optical Tweezers to Create Synthetic Tissue. Lab. Chip.

[12] Wells T., Schmidt H., Hawkins A. (2023). Nano/Microfluidic Device for High-Throughput Passive Trapping of Nanoparticles. Biomicrofluidics.

[13] Mohtar M.N., Abdulhameed A., Halin I.A., Hamidon M.N. (2020). Carbon Nanotube Collections by Electro-Osmosis in Microfluidic Systems. AIP Conf. Proc..

[14] Fu L.-M., Hou H.-H., Chiu P.-H., Yang R.-J. (2018). Sample Preconcentration from Dilute Solutions on Micro/Nanofluidic Platforms: A Review. Electrophoresis.

[15] Haywood D.G., Saha-Shah A., Baker L.A., Jacobson S.C. (2015). Fundamental Studies of Nanofluidics: Nanopores, Nanochannels, and Nanopipets. Anal. Chem..

[16] Sajeesh P., Sen A.K. (2014). Particle Separation and Sorting in Microfluidic Devices: A Review. Microfluid. Nanofluidics.

[17] Cetin B., Özer M.B., Solmaz M.E. (2014). Microfluidic Bio-Particle Manipulation for Biotechnology. Biochem. Eng. J..

[18] Xie Y., Rufo J., Zhong R., Rich J., Li P., Leong K.W., Huang T.J. (2020). Microfluidic Isolation and Enrichment of Nanoparticles. ACS Nano.

[19] Paiè P., Zandrini T., Vázquez R.M., Osellame R., Bragheri F. (2018). Particle Manipulation by Optical Forces in Microfluidic Devices. Micromachines.

[20] Deng Y., Guo Y., Xu B. (2020). Recent Development of Microfluidic Technology for Cell Trapping in Single Cell Analysis: A Review. Processes.

[21] Fatoyinbo H.O., Li X., Li X., Zhou Y. (2021). 8—Microfluidic Devices for Cell Manipulation. Microfluidic Devices for Biomedical Applications (Second Edition).

[22] Gong L., Cretella A., Lin Y. (2023). Microfluidic Systems for Particle Capture and Release: A Review. Biosens. Bioelectron..

[23] Lapizco-Encinas B.H. (2019). On the Recent Developments of Insulator-based Dielectrophoresis: A Review. Electrophoresis.

[24] Yin C., Jiang X., Mann S., Tian L., Drinkwater B.W. (2023). Acoustic Trapping: An Emerging Tool for Microfabrication Technology. Small.

[25] Squires T.M., Quake S.R. (2005). Microfluidics: Fluid Physics at the Nanoliter Scale. Rev. Mod. Phys..

[26] Tabeling P. (2023). Introduction to Microfluidics.

[27] Bocquet L., Charlaix E. (2010). Nanofluidics, from Bulk to Interfaces. Chem. Soc. Rev..

[28] Turner A.P.F. (2013). Biosensors: Sense and Sensibility. Chem. Soc. Rev..

[29] Mehrotra P. (2016). Biosensors and Their Applications—A Review. J. Oral Biol. Craniofacial Res..

[30] Schmid G. (2011). Nanoparticles: From Theory to Application.

[31] Mohanraj V.J., Chen Y. (2006). Nanoparticles—A Review. Trop. J. Pharm. Res..

[32] Ahn J., Ko J., Lee S., Yu J., Kim Y., Jeon N.L. (2018). Microfluidics in Nanoparticle Drug Delivery; From Synthesis to Pre-Clinical Screening. Adv. Drug Deliv. Rev..

[33] Walker Z.J., Wells T., Belliston E., Romney S., Walker S.B., Sampad M.J.N., Saiduzzaman S.M., Losakul R., Schmidt H., Hawkins A.R. (2022). Optofluidic Particle Manipulation Platform with Nanomembrane. Micromachines.

[34] Walker Z.J., Wells T., Belliston E., Walker S.B., Zeller C., Sampad M.J.N., Saiduzzaman S.M., Schmidt H., Hawkins A.R. (2022). Optofluidic Particle Manipulation: Optical Trapping in a Thin-Membrane Microchannel. Biosensors.

[35] Melzer J.E., McLeod E. (2018). Fundamental Limits of Optical Tweezer Nanoparticle Manipulation Speeds. ACS Nano.

[36] Hur S.C., Mach A.J., Di Carlo D. (2011). High-Throughput Size-Based Rare Cell Enrichment Using Microscale Vortices. Biomicrofluidics.

[37] Kumar S., Xuan J., Lee M.L., Tolley H.D., Hawkins A.R., Woolley A.T. (2013). Thin-Film Microfabricated Nanofluidic Arrays for Size-Selective Protein Fractionation. Lab. Chip.

[38] Kim J., Erath J., Rodriguez A., Yang C. (2014). A High-Efficiency Microfluidic Device for Size-Selective Trapping and Sorting. Lab. Chip.

[39] Zheng S., Lin H., Liu J.-Q., Balic M., Datar R., Cote R.J., Tai Y.-C. (2007). Membrane Microfilter Device for Selective Capture, Electrolysis and Genomic Analysis of Human Circulating Tumor Cells. J. Chromatogr. A.

[40] Wlodkowic D., Faley S., Zagnoni M., Wikswo J.P., Cooper J.M. (2009). Microfluidic Single-Cell Array Cytometry for the Analysis of Tumor Apoptosis. Anal. Chem..

[41] Tayebi M., Zhou Y., Tripathi P., Chandramohanadas R., Ai Y. (2020). Exosome Purification and Analysis Using a Facile Microfluidic Hydrodynamic Trapping Device. Anal. Chem..

[42] Tan W.-H., Takeuchi S. (2007). A Trap-and-Release Integrated Microfluidic System for Dynamic Microarray Applications. Proc. Natl. Acad. Sci. USA.

[43] Hamblin M.N., Xuan J., Maynes D., Tolley H.D., Belnap D.M., Woolley A.T., Lee M.L., Hawkins A.R. (2009). Selective Trapping and Concentration of Nanoparticles and Viruses in Dual-Height Nanofluidic Channels. Lab. Chip.

[44] Xuan J., Hamblin M.N., Stout J.M., Tolley H.D., Maynes R.D., Woolley A.T., Hawkins A.R., Lee M.L. (2011). Surfactant Addition and Alternating Current Electrophoretic Oscillation during Size Fractionation of Nanoparticles in Channels with Two or Three Different Height Segments. J. Chromatogr. A.

[45] Stout J.M., Johnson J.E., Kumar S., Woolley A.T., Hawkins A.R. Particle Trapping in Electrostatically Actuated Nanofluidic Barriers. Proceedings of the 2015 IEEE 58th International Midwest Symposium on Circuits and Systems (MWSCAS).

[46] Tonomura W., Tsutsui M., Arima A., Yokota K., Taniguchi M., Washio T., Kawai T. (2019). High-Throughput Single-Particle Detections Using a Dual-Height-Channel-Integrated Pore. Lab. Chip.

[47] Han J.Y., Yeh M., DeVoe D.L. (2023). Nanogap Traps for Passive Bacteria Concentration and Single-Point Confocal Raman Spectroscopy. Biomicrofluidics.

[48] Bruus H. (2007). Theoretical Microfluidics.

[49] Sollier E., Go D.E., Che J., Gossett D.R., O’Byrne S., Weaver W.M., Kummer N., Rettig M., Goldman J., Nickols N. (2014). Size-Selective Collection of Circulating Tumor Cells Using Vortex Technology. Lab. Chip.

[50] Raihan M.K., Li D., Kummetz A.J., Song L., Yu L., Xuan X. (2020). Vortex Trapping and Separation of Particles in Shear Thinning Fluids. Appl. Phys. Lett..

[51] Shen F., Li Z., Xue S., Li M., Liu Z. (2020). Particle Recirculating Orbits within Microvortices Using Microfluidics. J. Phys. Appl. Phys..

[52] Shen F., Gao J., Ai M., Li Z., Liu Z. (2023). Mechanism of Particle Dual-Orbital Motion in a Laminar Microvortex. Phys. Fluids.

[53] Kwon T., Jeon H., Hamel J.-F.P., Han J. (2024). Removal of Cell Clusters from CHO Suspension Cultures Based on Large-Particle Trapping Effect in Spiral Inertial Microfluidics. Sep. Purif. Technol..

[54] Ashkin A., Dziedzic J.M. (1971). Optical Levitation by Radiation Pressure. Appl. Phys. Lett..

[55] Ashkin A., Dziedzic J.M., Bjorkholm J.E., Chu S. (1986). Observation of a Single-Beam Gradient Force Optical Trap for Dielectric Particles. Opt. Lett..

[56] Cai H., Leake K.D., Schmidt H., De La Rue R., Herzig H.P., Gerken M. (2020). Planar Optofluidics for On-Chip Particle Manipulation. Biomedical Optical Sensors: Differentiators for Winning Technologies.

[57] Werner M., Merenda F., Piguet J., Salathé R.-P., Vogel H. (2011). Microfluidic Array Cytometer Based on Refractive Optical Tweezers for Parallel Trapping, Imaging and Sorting of Individual Cells. Lab. Chip.

[58] Mandal S., Serey X., Erickson D. (2010). Nanomanipulation Using Silicon Photonic Crystal Resonators. Nano Lett..

[59] Renaut C., Dellinger J., Cluzel B., Honegger T., Peyrade D., Picard E., de Fornel F., Hadji E. (2012). Assembly of Microparticles by Optical Trapping with a Photonic Crystal Nanocavity. Appl. Phys. Lett..

[60] Kang J.-H., Kim K., Ee H.-S., Lee Y.-H., Yoon T.-Y., Seo M.-K., Park H.-G. (2011). Low-Power Nano-Optical Vortex Trapping via Plasmonic Diabolo Nanoantennas. Nat. Commun..

[61] Kawata S., Tani T. (1996). Optically Driven Mie Particles in an Evanescent Field along a Channeled Waveguide. Opt. Lett..

[62] Hellesø O.G., Løvhaugen P., Subramanian A.Z., Wilkinson J.S., Ahluwalia B.S. (2012). Surface Transport and Stable Trapping of Particles and Cells by an Optical Waveguide Loop. Lab. Chip.

[63] Sergides M., Truong V.G., Chormaic S.N. (2016). Highly Tunable Plasmonic Nanoring Arrays for Nanoparticle Manipulation and Detection. Nanotechnology.

[64] Kühn S., Lunt E.J., Phillips B.S., Hawkins A.R., Schmidt H. (2009). Optofluidic Particle Concentration by a Long-Range Dual-Beam Trap. Opt. Lett..

[65] Kühn S., Phillips B.S., Lunt E.J., Hawkins A.R., Schmidt H. (2010). Ultralow Power Trapping and Fluorescence Detection of Single Particles on an Optofluidic Chip. Lab. Chip.

[66] Rahman M., Stott M.A., Harrington M., Li Y., Sampad M.J.N., Lancaster L., Yuzvinsky T.D., Noller H.F., Hawkins A.R., Schmidt H. (2019). On Demand Delivery and Analysis of Single Molecules on a Programmable Nanopore-Optofluidic Device. Nat. Commun..

[67] Rahman M., Harrington M., Stott M.A., Li Y., Sampad M.J.N., Yuzvinsky T.D., Hawkins A.R., Schmidt H. (2019). Optical Trapping Assisted Detection Rate Enhancement of Single Molecules on a Nanopore Optofluidic Chip. Optica.

[68] Sampad M.J.N., Zhang H., Yuzvinsky T.D., Stott M.A., Hawkins A.R., Schmidt H. (2021). Optical Trapping Assisted Label-Free and Amplification-Free Detection of SARS-CoV-2 RNAs with an Optofluidic Nanopore Sensor. Biosens. Bioelectron..

[69] Morgan H., Hughes M.P., Green N.G. (1999). Separation of Submicron Bioparticles by Dielectrophoresis. Biophys. J..

[70] Pohl H.A. (1951). The Motion and Precipitation of Suspensoids in Divergent Electric Fields. J. Appl. Phys..

[71] Huang Y., Pethig R. (1991). Electrode Design for Negative Dielectrophoresis. Meas. Sci. Technol..

[72] Schnelle T., Hagedorn R., Fuhr G., Fiedler S., Müller T. (1993). Three-Dimensional Electric Field Traps for Manipulation of Cells—Calculation and Experimental Verification. Biochim. Biophys. Acta BBA Gen. Subj..

[73] Müller T., Gerardino A., Schnelle T., Shirley S.G., Bordoni F., De Gasperis G., Leoni R., Fuhr G. (1996). Trapping of Micrometre and Sub-Micrometre Particles by High-Frequency Electric Fields and Hydrodynamic Forces. J. Phys. Appl. Phys..

[74] Müller T., Fiedler S., Schnelle T., Ludwig K., Jung H., Fuhr G. (1996). High Frequency Electric Fields for Trapping of Viruses. Biotechnol. Technol..

[75] Schnelle T., Muller T., Kentsch J., Grom F., Stelzle M. (2011). Method and Device for Collecting Suspended Particles. U.S. Patent.

[76] Hughes M.P., Hoettges F., Wattingham R. (2014). Device for Dielectrophoretic Manipulation of Particles. U.S. Patent.

[77] Hughes M.P. (2009). Apparatus for Collecting Particles 2009. U.S. Patent.

[78] Masuda S., Washizu M., Nanba T. (1989). Novel Method of Cell Fusion in Field Constriction Area in Fluid Integration Circuit. IEEE Trans. Ind. Appl..

[79] Ogle B.M., Cascalho M., Platt J.L. (2005). Biological Implications of Cell Fusion. Nat. Rev. Mol. Cell Biol..

[80] Nakidde D., Zellner P., Alemi M.M., Shake T., Hosseini Y., Riquelme M.V., Pruden A., Agah M. (2015). Three Dimensional Passivated-Electrode Insulator-Based Dielectrophoresis. Biomicrofluidics.

[81] Chiou C.-H., Chien L.-J., Kuo J.-N. (2015). Nanoconstriction-Based Electrodeless Dielectrophoresis Chip for Nanoparticle and Protein Preconcentration. Appl. Phys. Express.

[82] Cummings E.B., Singh A.K. (2003). Dielectrophoresis in Microchips Containing Arrays of Insulating Posts:  Theoretical and Experimental Results. Anal. Chem..

[83] Lapizco-Encinas B.H., Simmons B.A., Cummings E.B., Fintschenko Y. (2004). Insulator-based Dielectrophoresis for the Selective Concentration and Separation of Live Bacteria in Water. Electrophoresis.

[84] Chen D., Du H. (2010). A Microfluidic Device for Rapid Concentration of Particles in Continuous Flow by DC Dielectrophoresis. Microfluid. Nanofluidics.

[85] Hoettges K.F., Hughes M.P., Cotton A., Hopkins N.A.E., McDonnell M.B. (2003). Optimizing Particle Collection for Enhanced Surface-Based Biosensors. IEEE Eng. Med. Biol. Mag..

[86] Mohtar M.N., Hoettges K.F., Hughes M.P. (2014). Factors Affecting Particle Collection by Electro-Osmosis in Microfluidic Systems. Electrophoresis.

[87] Hübner Y., Hoettges K.F., McDonnell M.B., Carter M.J., Hughes M.P. (2007). Applications of Dielectrophoretic/Electro-Hydrodynamic “Zipper” Electrodes for Detection of Biological Nanoparticles. Int. J. Nanomed..

[88] Wong P.K., Chen C.-Y., Wang T.-H., Ho C.-M. An AC Electroosmotic Processor for Biomolecules. Proceedings of the TRANSDUCERS ’03. 12th International Conference on Solid-State Sensors, Actuators and Microsystems. Digest of Technical Papers (Cat. No.03TH8664).

[89] Wong P.K., Chen C.-Y., Wang T.-H., Ho C.-M. (2004). Electrokinetic Bioprocessor for Concentrating Cells and Molecules. Anal. Chem..

[90] Gagnon Z., Chang H.-C. (2005). Aligning Fast Alternating Current Electroosmotic Flow Fields and Characteristic Frequencies with Dielectrophoretic Traps to Achieve Rapid Bacteria Detection. Electrophoresis.

[91] Cheng I.-F., Chang H.-C., Chen T.-Y., Hu C., Yang F.-L. (2013). Rapid (<5 Min) Identification of Pathogen in Human Blood by Electrokinetic Concentration and Surface-Enhanced Raman Spectroscopy. Sci. Rep..

[92] Park S., Koklu M., Beskok A. (2009). Particle Trapping in High-Conductivity Media with Electrothermally Enhanced Negative Dielectrophoresis. Anal. Chem..

[93] Electrokinetic Merriam-Webstercom Online Dict. https://www.merriam-webster.com/dictionary/electrokinetic.

[94] Xuan X. (2019). Recent Advances in Direct Current Electrokinetic Manipulation of Particles for Microfluidic Applications. Electrophoresis.

[95] Song Y., Chen P., Chung M.T., Nidetz R., Park Y., Liu Z., McHugh W., Cornell T.T., Fu J., Kurabayashi K. (2017). AC Electroosmosis-Enhanced Nanoplasmofluidic Detection of Ultralow-Concentration Cytokine. Nano Lett..

[96] Wu J., Ben Y., Battigelli D., Chang H.-C. (2005). Long-Range AC Electroosmotic Trapping and Detection of Bioparticles. Ind. Eng. Chem. Res..

[97] Wu J., Ben Y., Chang H.-C. (2005). Particle Detection by Electrical Impedance Spectroscopy with Asymmetric-Polarization AC Electroosmotic Trapping. Microfluid. Nanofluidics.

[98] Bhatt K.H., Grego S., Velev O.D. (2005). An AC Electrokinetic Technique for Collection and Concentration of Particles and Cells on Patterned Electrodes. Langmuir.

[99] Hou D., Maheshwari S., Chang H.-C. (2007). Rapid Bioparticle Concentration and Detection by Combining a Discharge Driven Vortex with Surface Enhanced Raman Scattering. Biomicrofluidics.

[100] Dey R., Shaik V.A., Chakraborty D., Ghosal S., Chakraborty S. (2015). AC Electric Field-Induced Trapping of Microparticles in Pinched Microconfinements. Langmuir.

[101] Yang K., Wu J. In Situ Electrokinetic Preconcentrator for Conductive Biofluids. Proceedings of the American Society of Mechanical Engineers Digital Collection.

[102] Yang K., Wu J. (2010). Numerical Study of in Situ Preconcentration for Rapid and Sensitive Nanoparticle Detection. Biomicrofluidics.

[103] Sun H., Ren Y., Tao Y., Jiang T., Jiang H. (2021). Flexible Online In-Droplet Cell/Synthetic Particle Concentration Utilizing Alternating Current Electrothermal-Flow Field-Effect Transistor. Lab. Chip.

[104] Abdelghany A., Ichikawa Y., Motosuke M. (2023). Tuning AC Electrokinetic Flow to Enhance Nanoparticle Accumulation in Low-Conductivity Solutions. Adv. Mater. Interfaces.

[105] Richetti P., Prost J., Barois P. (1984). Two-Dimensional Aggregation and Crystallization of a Colloidal Suspension of Latex Spheres. J. Phys. Lett..

[106] Trau M., Saville D.A., Aksay I.A. (1996). Field-Induced Layering of Colloidal Crystals. Science.

[107] Trau M., Saville D.A., Aksay I.A. (1997). Assembly of Colloidal Crystals at Electrode Interfaces. Langmuir.

[108] Williams S.J., Kumar A., Wereley S.T. (2008). Electrokinetic Patterning of Colloidal Particles with Optical Landscapes. Lab. Chip.

[109] Velasco V., Williams S.J. (2013). Electrokinetic Concentration, Patterning, and Sorting of Colloids with Thin Film Heaters. J. Colloid Interface Sci..

[110] Guan W., Park J.H., Krstić P.S., Reed M.A. (2011). Non-Vanishing Ponderomotive AC Electrophoretic Effect for Particle Trapping. Nanotechnology.

[111] Aïzel K., Fouillet Y., Pudda C. (2014). Electropreconcentration of Nanoparticles Using a Radial Micro-Nanofluidic Device. J. Nanoparticle Res..

[112] Tarn M.D., Peyman S.A., Pamme N. (2013). Simultaneous Trapping of Magnetic and Diamagnetic Particle Plugs for Separations and Bioassays. RSC Adv..

[113] Watarai H., Namba M. (2002). Capillary Magnetophoresis of Human Blood Cells and Their Magnetophoretic Trapping in a Flow System. J. Chromatogr. A.

[114] Hejazian M., Nguyen N.-T. (2016). Magnetofluidic Concentration and Separation of Non-Magnetic Particles Using Two Magnet Arrays. Biomicrofluidics.

[115] Kimura T., Sato Y., Kimura F., Iwasaka M., Ueno S. (2005). Micropatterning of Cells Using Modulated Magnetic Fields. Langmuir.

[116] Kimura T., Yamato M., Nara A. (2004). Particle Trapping and Undulation of a Liquid Surface Using a Microscopically Modulated Magnetic Field. Langmuir.

[117] Ramadan Q., Samper V., Poenar D.P., Yu C. (2006). An Integrated Microfluidic Platform for Magnetic Microbeads Separation and Confinement. Biosens. Bioelectron..

[118] Gooneratne C.P., Liang C., Giouroudi I., Kosel J. (2011). A Magnetic Particle Micro-Trap for Large Trapping Surfaces. Procedia Eng..

[119] Gooneratne C.P., Giouroudi I., Liang C., Kosel J. (2011). A Giant Magnetoresistance Ring-Sensor Based Microsystem for Magnetic Bead Manipulation and Detection. J. Appl. Phys..

[120] Li F., Kodzius R., Gooneratne C.P., Foulds I.G., Kosel J. (2014). Magneto-Mechanical Trapping Systems for Biological Target Detection. Microchim. Acta.

[121] Gooneratne C.P., Kosel J. A Micro-Pillar Array to Trap Magnetic Beads in Microfluidic Systems. Proceedings of the 2012 Sixth International Conference on Sensing Technology (ICST).

[122] Yu X., He R., Li S., Cai B., Zhao L., Liao L., Liu W., Zeng Q., Wang H., Guo S.-S. (2013). Magneto-Controllable Capture and Release of Cancer Cells by Using a Micropillar Device Decorated with Graphite Oxide-Coated Magnetic Nanoparticles. Small.

[123] Faivre M., Gelszinnis R., Degouttes J., Terrier N., Rivière C., Ferrigno R., Deman A.-L. (2014). Magnetophoretic Manipulation in Microsystem Using Carbonyl Iron-Polydimethylsiloxane Microstructures. Biomicrofluidics.

[124] Smistrup K., Bruus H., Hansen M.F. (2007). Towards a Programmable Magnetic Bead Microarray in a Microfluidic Channel. J. Magn. Magn. Mater..

[125] Lefebvre O., Cao H.H., Cortés Francisco M., Woytasik M., Dufour-Gergam E., Ammar M., Martincic E. (2020). Reusable Embedded Microcoils for Magnetic Nano-Beads Trapping in Microfluidics: Magnetic Simulation and Experiments. Micromachines.

[126] Song S.-H., Kwak B.-S., Park J.-S., Kim W., Jung H.-I. (2009). Novel Application of Joule Heating to Maintain Biocompatible Temperatures in a Fully Integrated Electromagnetic Cell Sorting System. Sens. Actuators Phys..

[127] Zheng Y., Sawan M. (2013). Planar Microcoil Array Based Temperature-Controllable Lab-on-Chip Platform. IEEE Trans. Magn..

[128] Bücks K., Müller H. (1933). Über einige Beobachtungen an schwingenden Piezoquarzen und ihrem Schallfeld. Z. Für Phys..

[129] Sarvazyan A.P., Rudenko O.V., Fatemi M. (2021). Acoustic Radiation Force: A Review of Four Mechanisms for Biomedical Applications. IEEE Trans. Ultrason. Ferroelectr. Freq. Control.

[130] Lilliehorn T., Nilsson M., Simu U., Johansson S., Almqvist M., Nilsson J., Laurell T. (2005). Dynamic Arraying of Microbeads for Bioassays in Microfluidic Channels. Sens. Actuators B Chem..

[131] Shilton R., Tan M.K., Yeo L.Y., Friend J.R. (2008). Particle Concentration and Mixing in Microdrops Driven by Focused Surface Acoustic Waves. J. Appl. Phys..

[132] Raghavan R.V., Friend J.R., Yeo L.Y. (2010). Particle Concentration via Acoustically Driven Microcentrifugation: microPIV Flow Visualization and Numerical Modelling Studies. Microfluid. Nanofluidics.

[133] Rogers P.R., Friend J.R., Yeo L.Y. (2010). Exploitation of Surface Acoustic Waves to Drive Size-Dependent Microparticle Concentration within a Droplet. Lab. Chip.

[134] Destgeer G., Cho H., Hang Ha B., Ho Jung J., Park J., Jin Sung H. (2016). Acoustofluidic Particle Manipulation inside a Sessile Droplet: Four Distinct Regimes of Particle Concentration. Lab. Chip.

[135] Whitehill J., Neild A., Ng T.W., Stokes M. (2010). Collection of Suspended Particles in a Drop Using Low Frequency Vibration. Appl. Phys. Lett..

[136] Hammarström B., Laurell T., Nilsson J. (2012). Seed Particle-Enabled Acoustic Trapping of Bacteria and Nanoparticles in Continuous Flow Systems. Lab. Chip.

[137] Evander M., Gidlöf O., Olde B., Erlinge D., Laurell T. (2015). Non-Contact Acoustic Capture of Microparticles from Small Plasma Volumes. Lab. Chip.

[138] Cui W., Mu L., Duan X., Pang W., Reed M.A. (2019). Trapping of Sub-100 Nm Nanoparticles Using Gigahertz Acoustofluidic Tweezers for Biosensing Applications. Nanoscale.

[139] Zhou Y., Ma Z., Ai Y. (2020). Submicron Particle Concentration and Patterning with Ultralow Frequency Acoustic Vibration. Anal. Chem..

[140] Fakhfouri A., Devendran C., Collins D.J., Ai Y., Neild A. (2016). Virtual Membrane for Filtration of Particles Using Surface Acoustic Waves (SAW). Lab. Chip.

[141] Collins D.J., Luan Khoo B., Ma Z., Winkler A., Weser R., Schmidt H., Han J., Ai Y. (2017). Selective Particle and Cell Capture in a Continuous Flow Using Micro-Vortex Acoustic Streaming. Lab. Chip.

[142] Kane R.S., Takayama S., Ostuni E., Ingber D.E., Whitesides G.M. (1999). Patterning Proteins and Cells Using Soft Lithography. Biomaterials.

[143] Dharmasiri U., Njoroge S.K., Witek M.A., Adebiyi M.G., Kamande J.W., Hupert M.L., Barany F., Soper S.A. (2011). High-Throughput Selection, Enumeration, Electrokinetic Manipulation, and Molecular Profiling of Low-Abundance Circulating Tumor Cells Using a Microfluidic System. Anal. Chem..

[144] Xu Y., Phillips J.A., Yan J., Li Q., Fan Z.H., Tan W. (2009). Aptamer-Based Microfluidic Device for Enrichment, Sorting, and Detection of Multiple Cancer Cells. Anal. Chem..

[145] Mu X., Zheng W., Sun J., Zhang W., Jiang X. (2013). Microfluidics for Manipulating Cells. Small.

[146] Sigurdson M., Meinhart C., Wang D., Liu X., Feng J.J., Krishnamoorthy S., Sundaram S. AC Electrokinetics for Microfluidic Immunosensors. Proceedings of the American Society of Mechanical Engineers Digital Collection.

[147] Syed A., Mangano L., Mao P., Han J., Song Y.-A. (2014). Creating Sub-50 Nm Nanofluidic Junctions in a PDMS Microchip via Self-Assembly Process of Colloidal Silica Beads for Electrokinetic Concentration of Biomolecules. Lab. Chip.

[148] Gerspach M.A., Mojarad N., Sharma D., Ekinci Y., Pfohl T. (2018). Pneumatically Controlled Nanofluidic Devices for Contact-Free Trapping and Manipulation of Nanoparticles. Part. Part. Syst. Charact..

[149] Krafft B., Tycova A., Urban R.D., Dusny C., Belder D. (2021). Microfluidic Device for Concentration and SERS-based Detection of Bacteria in Drinking Water. Electrophoresis.

[150] Habibi R., Neild A. (2019). Sound Wave Activated Nano-Sieve (SWANS) for Enrichment of Nanoparticles. Lab. Chip.

[151] Allahrabbi N., Chia Y.S.M., Saifullah M.S.M., Lim K.-M., Yung L.Y.L. (2015). A Hybrid Dielectrophoretic System for Trapping of Microorganisms from Water. Biomicrofluidics.

[152] Kumar A., Williams S.J., Chuang H.S., Green N.G., Wereley S.T. (2011). Hybrid Opto-Electric Manipulation in Microfluidics—Opportunities and Challenges. Lab. Chip.

[153] Al-Ali A., Waheed W., Abu-Nada E., Alazzam A. (2022). A Review of Active and Passive Hybrid Systems Based on Dielectrophoresis for the Manipulation of Microparticles. J. Chromatogr. A.

[154] Niculescu A.-G., Chircov C., Bîrcă A.C., Grumezescu A.M. (2021). Fabrication and Applications of Microfluidic Devices: A Review. Int. J. Mol. Sci..

[155] Baron V.O., Chen M., Hammarstrom B., Hammond R.J.H., Glynne-Jones P., Gillespie S.H., Dholakia K. (2020). Real-Time Monitoring of Live Mycobacteria with a Microfluidic Acoustic-Raman Platform. Commun. Biol..

[156] Branton D., Deamer D.W., Marziali A., Bayley H., Benner S.A., Butler T., Di Ventra M., Garaj S., Hibbs A., Huang X. (2008). The Potential and Challenges of Nanopore Sequencing. Nat. Biotechnol..

[157] Howorka S., Siwy Z. (2009). Nanopore Analytics: Sensing of Single Molecules. Chem. Soc. Rev..

[158] Liu Q., Wu H., Wu L., Xie X., Kong J., Ye X., Liu L. (2012). Voltage-Driven Translocation of DNA through a High Throughput Conical Solid-State Nanopore. PLoS ONE.

[159] Yuan Z., Liu Y., Dai M., Yi X., Wang C. (2020). Controlling DNA Translocation Through Solid-State Nanopores. Nanoscale Res. Lett..

[160] Schmidt H., Sampad M., Saiduzzaman M.S., Hawkins A.R., Walker Z., Wells T., Dholakia K., Spalding G.C. (2022). Recent Advances in Waveguide-Based Optical Trapping for Molecular Biomarker Analysis. Proceedings of the Optical Trapping and Optical Micromanipulation XIX.

[161] Zhang L., Tian Z., Bachman H., Zhang P., Huang T.J. (2020). A Cell-Phone-Based Acoustofluidic Platform for Quantitative Point-of-Care Testing. ACS Nano.

[162] Grünberger A., Paczia N., Probst C., Schendzielorz G., Eggeling L., Noack S., Wiechert W., Kohlheyer D. (2012). A Disposable Picolitre Bioreactor for Cultivation and Investigation of Industrially Relevant Bacteria on the Single Cell Level. Lab. Chip.

[163] Hage D.S. (1999). Immunoassays. Anal. Chem..

[164] Wisdom G.B. (1976). Enzyme-Immunoassay. Clin. Chem..

[165] Nienhaus G.U., Nienhaus K., Kubitscheck U. (2017). Fluorescence Labeling. Fluorescence Microscopy.

[166] Liu Y., Guo S., Zhang Z., Huang W., Baigl D., Xie M., Chen Y., Pang D. (2007). A Micropillar-integrated Smart Microfluidic Device for Specific Capture and Sorting of Cells. Electrophoresis.

[167] Garg N., Westerhof T.M., Liu V., Liu R., Nelson E.L., Lee A.P. (2018). Whole-Blood Sorting, Enrichment and in Situ Immunolabeling of Cellular Subsets Using Acoustic Microstreaming. Microsyst. Nanoeng..

[168] Jung D.R., Kapur R., Adams T., Giuliano K.A., Mrksich M., Craighead H.G., Taylor D.L. (2001). Topographical and Physicochemical Modification of Material Surface to Enable Patterning of Living Cells. Crit. Rev. Biotechnol..

[169] Garvin K.A., Hocking D.C., Dalecki D. (2010). Controlling the Spatial Organization of Cells and Extracellular Matrix Proteins in Engineered Tissues Using Ultrasound Standing Wave Fields. Ultrasound Med. Biol..

[170] Sharma B., Sharma A. (2022). Microfluidics: Recent Advances Toward Lab-on-Chip Applications in Bioanalysis. Adv. Eng. Mater..

[171] Mohammed M.I., Haswell S., Gibson I. (2015). Lab-on-a-Chip or Chip-in-a-Lab: Challenges of Commercialization Lost in Translation. Procedia Technol..

[172] Whitesides G.M. (2006). The Origins and the Future of Microfluidics. Nature.

